# Appearance anxiety and appearance-management behaviors on Chinese social media: evidence for prospective bidirectional associations

**DOI:** 10.3389/fpsyg.2026.1926306

**Published:** 2026-09-03

**Authors:** Dinghui Ma, Hourong Yu, Weijia Li, Yi Tang

**Affiliations:** Nanjing University of Science and Technology, School of Design, Art and Media, Nanjing, Jiangsu, China

**Keywords:** appearance anxiety, body image, Chinese platforms, cross-lagged panel model, fear of negative evaluation, social media, uses and gratifications

## Abstract

**Background:**

Research on appearance anxiety and social media use has predominantly treated social media exposure as the cause and appearance anxiety as the outcome. This study examined the reverse and bidirectional pathways: whether appearance anxiety is prospectively associated with three functionally distinct appearance-oriented social media behaviors, and whether these behaviors are associated with subsequent anxiety, in young Chinese women.

**Method:**

A two-wave panel design with a 6-month interval was employed with 339 young Chinese women (aged 18–35; 87.1% Wave-2 retention). Cross-lagged panel models (CLPM) estimated bidirectional prospective between-persons associations, with an assumption-dependent phantom-variable sensitivity analysis used only to assess robustness under fixed stable-variance assumptions. Fear of negative evaluation (FNE) was examined as a candidate dispositional link using a partially longitudinal indirect-association model (T1 anxiety -> T1 FNE -> T2 behavior, controlling for baseline behavior), since FNE was assessed only at baseline. Whether cognitive-level media literacy attenuated this indirect association was addressed as a research question.

**Results:**

Appearance anxiety was prospectively associated with all three T2 behaviors at the between-persons level (βs = 0.28–0.39, *p*s < 0.001), and reverse-direction paths were of comparable or larger magnitude (βs = 0.38–0.50); both directions held in sensitivity analyses. Findings were consistent with a modest indirect association through FNE (indirect effects = 0.057–0.095), smaller than the direct effects of anxiety (0.235–0.339). An exploratory extension testing media literacy as a moderator of the FNE-to-behavior link found a significant, counter-theoretical effect for beauty-filter use only.

**Conclusions:**

Appearance anxiety and appearance-oriented social media behaviors show prospective bidirectional between-persons associations in a Chinese platform ecology, robust to sensitivity checks. FNE may function as one candidate link between habitual appearance anxiety and downstream appearance-management behavior, though a reverse-order robustness check could not rule out FNE preceding anxiety. The present study did not find statistically reliable evidence that cognitive-level media literacy buffers this pathway, tentatively suggesting that effective intervention may need to target appearance-specific anxiety directly rather than relying solely on cognitive media literacy education. An exploratory finding that media literacy may strengthen, rather than weaken, the FNE-behavior link for beauty-filter use is counter-theoretical and warrants confirmatory replication before informing intervention design.

## 1 Introduction

With the rapid proliferation of social media platforms, appearance anxiety has emerged as an increasingly prominent mental health concern among young women. Scholars have observed that the commodification of women's bodies through digital media compels many individuals to pursue extreme measures—including cosmetic procedures and restrictive dieting—to address perceived appearance deficiencies (Fredrickson and Roberts, 1997).

Social media platforms have become deeply embedded in the daily lives of young women, serving as core sites for appearance-related discourse, social comparison, and self-presentation (Fardouly and Vartanian, 2015). In China, three platforms dominate this ecology: Xiaohongshu (Little Red Book), Douyin (TikTok's Chinese counterpart), and Weibo. Xiaohongshu's dual community-and-commerce architecture integrates beauty product recommendations directly into appearance content feeds, substantially narrowing the psychological distance between information-seeking and consumption. Douyin embeds AI-powered beautification filters at the point of video capture, normalizing real-time appearance enhancement and reducing the friction cost of filter use. Weibo's trending-topic mechanism amplifies collective appearance standards through widely circulated aesthetic challenges. Substantial evidence confirms significant associations between social media use and appearance anxiety and body dissatisfaction (McComb and Mills, 2022; Gurtala and Fardouly, 2023).

The dominant paradigm in extant research treats social media use as the independent variable and appearance anxiety as the outcome (Gurtala and Fardouly, 2023; McComb and Mills, 2022). Although this paradigm has accumulated rich evidence, it systematically overlooks a theoretically important and practically consequential reverse pathway: whether appearance anxiety itself is prospectively associated with specific types of social media use behavior. Uses-and-gratifications theory (UGT; Blumler and Katz, 1975) provides a theoretical rationale for this reverse logic: audiences actively select media experiences to satisfy pre-existing psychological needs. Appearance anxiety may constitute precisely such a need state, activating appearance-management motivations that may be associated with individuals' selection of particular social media behaviors. If these behaviors fail to provide durable satisfaction, they may in turn be associated with higher subsequent anxiety, consistent with a possible pattern of bidirectional prospective associations.

The present study proposes that appearance anxiety, as a persistent state of negative self-evaluation, is associated with individuals' selection of specific social media behaviors to manage anxiety, seek a sense of control, or obtain external validation; and that these behaviors are, in turn, associated with higher subsequent anxiety, consistent with a possible pattern of bidirectional prospective associations.

## 2 Literature review and research hypotheses

### 2.1 Social media use and appearance anxiety

Appearance anxiety refers to persistent anxiety arising from concern that one's physical appearance fails to meet socially prescribed aesthetic standards and fear of receiving negative evaluations from others (Dion et al., 1990). It is distinct from general anxiety in that it is specifically oriented toward the evaluative dimension of physical appearance (Boursier et al., 2020), and should be differentiated from related constructs such as body dissatisfaction and social appearance anxiety: body dissatisfaction emphasizes cognitive-affective evaluation of one's own body, whereas appearance anxiety foregrounds anticipatory concern about being judged by others on the basis of one's appearance (Seekis et al., 2020).

The impact of social media use on appearance anxiety has been supported by abundant empirical research. Experimental studies indicate that exposure to idealized appearance content is associated with lower women's satisfaction with their own appearance and greater internalization of ideal appearance standards (Gurtala and Fardouly, 2023). Studies employing controlled experimental designs similarly find that participants exposed to media-ideal body images report significantly lower satisfaction with their own weight and appearance relative to control groups (McComb and Mills, 2022). Regarding candidate indirect pathways, recent studies have constructed chain mediation models: for instance, research among young acne patients found that thin-ideal internalization and fear of negative evaluation serially mediated the association between social media information exposure and appearance anxiety (Zhang and Zhou, 2024). Other work, using Xiaohongshu as the focal platform, found that social media use influenced women's appearance anxiety via the chain mediation of thin-ideal internalization and appearance comparison, though the mediating role of self-objectification was non-significant (Liu, 2024).

Extant research has almost universally positioned social media use as the independent variable and appearance anxiety as the dependent variable. However, emerging findings begin to reveal the existence of a reverse relationship: one study found that, for adolescents and young adults, higher body dissatisfaction predicted seeking social comparison-type gratifications via social media, suggesting that body dissatisfaction and social comparison may be bidirectional. This finding directly challenges the assumption of unidirectional causality, yet systematic examination of the reverse pathway remains exceptionally rare.

### 2.2 A three-dimensional conceptualization of appearance-oriented social media behaviors

Prior research on appearance-related social media use has often aggregated diverse behaviors into a single index of use intensity or frequency, a practice that risks obscuring functionally important differences. The present study instead distinguishes three appearance-oriented behaviors on both conceptual and empirical grounds. Conceptually, the three behaviors differ along at least two dimensions relevant to appearance management: locus of control and temporal orientation. Beauty-filter use is self-directed and immediate, allowing individuals to directly and instantaneously alter their own presented image without depending on external information or validation. Appearance information-seeking is knowledge-oriented and comparatively delayed in its effects, involving the acquisition of improvement strategies (e.g., skincare routines, cosmetic procedures) whose benefits, if any, accrue gradually rather than immediately. Appearance feedback-seeking is other-directed and contingent, requiring the participation of an external audience and deferring gratification until others respond. Empirically, this three-dimensional structure receives direct support from the confirmatory factor analyses reported in Section 4.2: a three-factor model fit the data substantially better than two-factor or one-factor alternatives that collapsed these behaviors together, indicating that respondents' answers are not well-explained by a single underlying appearance-behavior factor. We therefore treat the three behaviors as distinct outcomes throughout, consistent with Katz et al.'s (1973) classification of media gratifications into functionally separable types (see Table 1).

**Table 1:** Theoretical classification of appearance-oriented social media behaviors: UGT gratification motivations and Chinese platform affordances.

Behavioral dimension	UGT gratification motivation	Psychological function	Chinese platform affordances
Beauty-filter use	Self-presentation management (self-identity reinforcement)	Reduce appearance-exposure risk; pre-emptively avert negative evaluation	Xiaohongshu AR beauty filters; Douyin AI beautification; Weibo beauty filter stickers
Appearance information-seeking	Information surveillance/acquisition	Bridge appearance knowledge gaps; acquire improvement strategies and methods	Xiaohongshu skincare/cosmetics notes; Douyin makeup tutorials; platform product recommendations
Appearance feedback-seeking	Social validation/approval	Obtain external confirmation of attractiveness; stabilize appearance-contingent self-worth	Post likes and comments; Xiaohongshu “endorsement” approval; Douyin interaction features

UGT functional types follow Katz et al. (1973). Platform affordance characteristics reflect conditions during the data collection period.

#### 2.2.1 Beauty-filter use: self-presentation management

Beauty-filter use refers to the behavior of choosing, editing, and posting photographs of oneself using beautification, smoothing, slimming, and other technical filters on social media (Fox and Vendemia, 2016). With the proliferation of photo-editing and filter applications, such behavior has become extremely common among young women; a UK survey found that approximately 90% of young women report using a filter or editing their photos before posting (Gill, 2021). Research shows that frequent photo-editing behavior is associated with greater body dissatisfaction (Tiggemann et al., 2020), and women who more frequently use photo-editing applications before posting selfies report greater fear of negative evaluation than those who rarely or never do so (Bugeja et al., 2026). Functionally, beauty-filter use can be understood as a form of selective self-presentation that narrows the gap between actual and idealized appearance (Fox and Vendemia, 2016).

**H1:** Controlling for T1 beauty-filter use, T1 appearance anxiety will positively predict T2 beauty-filter use.

#### 2.2.2 Appearance information-seeking: surveillance behavior

Appearance information-seeking refers to the behavior of actively searching for and browsing content related to skincare, cosmetics, outfit styling, cosmetic procedures, and body shaping on social media (Åberg et al., 2020). Individuals who are insecure about their appearance tend to actively seek such information in pursuit of routes to appearance improvement (Mitchell and Grieve, 2020). Research finds that attention to appearance-related content is associated with elevated levels of appearance comparison and thin-ideal internalization, and that individuals who evaluate their own appearance critically are more inclined to use social media as a channel for information acquisition and improvement strategies (Wang et al., 2023). Unlike the “immediate modification” afforded by beauty filters, appearance information-seeking is oriented toward longer-term, information-acquisition coping.

**H2:** Controlling for T1 appearance information-seeking, T1 appearance anxiety will positively predict T2 appearance information-seeking.

#### 2.2.3 Appearance feedback-seeking: social validation

Appearance feedback-seeking refers to the behavior of publishing appearance-related content on social media and attending to the resulting likes and comments so as to confirm one's attractiveness and obtain appearance-related approval (Kvardova et al., 2023). Research shows that individuals who place high value on online validation such as likes and comments are more susceptible to self-esteem fluctuation and body dissatisfaction (Rousseau et al., 2020), and those who evaluate their own appearance harshly are more inclined to seek appearance-related approval on social media, with this feedback-driven cycle potentially further intensifying appearance anxiety (Gurtala and Fardouly, 2023). Functionally, appearance feedback-seeking fulfills the need for external validation; individuals high in appearance anxiety lack confidence in their own appearance and turn to others' positive feedback for confirmation and reassurance (Jin and Le, 2024).

**H3:** Controlling for T1 appearance feedback-seeking, T1 appearance anxiety will positively predict T2 appearance feedback-seeking.

### 2.3 Theoretical basis: integrating uses-and-gratifications theory with complementary perspectives

The present account draws primarily on UGT, but UGT is best understood as complementary to, rather than a replacement for, established frameworks that explain why social media exposure generates appearance anxiety in the first place. Objectification theory (Fredrickson and Roberts, 1997) proposes that a sociocultural milieu that habitually treats the female body as an object to be visually evaluated fosters self-objectification and appearance-related self-surveillance, which in turn generate anxiety, shame, and diminished wellbeing. Social comparison theory (Festinger, 1954), as extended to the appearance domain, holds that individuals evaluate their own appearance by comparing it to available social referents; the abundance of idealized, algorithmically curated appearance content on social media affords frequent upward comparisons that elevate appearance-related distress. Self-discrepancy theory (Higgins, 1987) offers a complementary account of the resulting emotional experience: discrepancies between one's actual self and one's ought self (i.e., how one believes one should appear, including to meet others' expectations) are theorized to produce agitation-related emotions such as anxiety and apprehension, whereas actual-ideal discrepancies are more closely tied to dejection-related emotions such as sadness. Applied to appearance, this suggests that appearance anxiety may be particularly linked to a perceived gap between one's actual and one's socially expected appearance, a gap that exposure to idealized social media content may widen. These three frameworks converge on explaining the well-established forward pathway from social media exposure to appearance anxiety (reviewed in Section 2.1), but are largely silent on the reverse and dynamic questions this study addresses: once appearance anxiety is elicited, which specific media behaviors does it motivate, and could those behaviors feed back into subsequent anxiety? UGT is theoretically suited to this latter question because, unlike the frameworks above, it explicitly models media use as a motivated, need-driven selection process rather than as a passive environmental exposure. The present study accordingly treats appearance anxiety—itself explicable via objectification, social comparison, or self-discrepancy processes—as a need state that, per UGT, selectively activates specific appearance-management behaviors, integrating these complementary perspectives rather than relying on UGT in isolation.

UGT (Blumler and Katz, 1975; Palmgreen and Rayburn, 1982) holds that individuals actively select media experiences to fulfill identifiable psychological needs. Within the UGT framework, the distinction between gratifications sought and gratifications obtained is particularly important (Palmgreen and Rayburn, 1982). Individuals' expectancy-value beliefs about what gratifications a given medium can provide (e.g., “beauty filters will make me look better,” “searching skincare content will help me improve my skin”) may be associated with specific media use behaviors. This expectancy-value perspective provides a rationale for expecting appearance anxiety, as a psychological need state, to be associated with specific behavioral choices: higher anxiety may correspond to stronger expectations that relevant media behaviors can satisfy needs for control and validation, and thus to more frequent corresponding behaviors (Dingemans et al., 2023). Whether through managing self-presentation via beauty filters, acquiring improvement methods through information-seeking, or obtaining external validation through feedback-seeking, the present study explicitly theorizes the motivational layer: appearance anxiety (need state) activates three functionally distinct appearance-management motivations, each corresponding to one of three measurable behaviors (see Table 1). Self-presentation management motivation may be associated with beauty-filter use; information surveillance motivation may be associated with appearance information-seeking; social validation motivation may be associated with appearance feedback-seeking. The three behavioral dimensions correspond respectively to media function types identified by Katz et al. (1973): personal identity/self-reinforcement, information acquisition, and social integration.

It is also worth situating this motivational account against plausible alternative explanations for the same predicted associations. First, a third-variable account is possible: stable individual differences such as trait negative affectivity or neuroticism could jointly elevate both appearance anxiety and general social media engagement, producing an anxiety-behavior association that does not reflect motivated appearance-management selection at all. Second, a general engagement account would attribute the predicted associations to overall social media use intensity rather than to the appearance-specific gratification-seeking process UGT proposes; under this account, heavier social media users would show elevated anxiety and elevated appearance-behavior engagement simply because they are exposed to more content of every kind, not because anxiety selectively motivates appearance-related behavior. Third, a purely reactive account would hold that anxious individuals engage in these behaviors as an unreflective, habitual response to negative affect rather than as the expectancy-value-driven selection UGT assumes. The present design cannot fully adjudicate between these accounts and the proposed UGT-based explanation, since it does not measure trait negative affectivity, general (non-appearance) social media use, or the expectancy-value beliefs UGT's mechanism presupposes; we return to this limitation in Section 5.5. We note, however, that the three behaviors under study are appearance-specific rather than general engagement metrics, and that the CLPM models control for baseline levels of the outcome, age, education, and BMI, which partially—though not fully—constrains competing third-variable explanations.

### 2.4 Candidate indirect pathway: fear of negative evaluation

Fear of negative evaluation (FNE) refers to individuals' apprehension and dread regarding receiving negative evaluations from others in social situations (Carleton et al., 2011). The present study examines FNE as a candidate indirect pathway linking appearance anxiety to appearance-oriented social media behaviors.

The theoretical logic is as follows: individuals high in appearance anxiety fundamentally lack confidence that their appearance will pass others' evaluative scrutiny, rendering them more susceptible to fear of negative evaluation. Extant research shows that appearance anxiety and FNE are closely associated, and that appearance anxiety significantly positively predicts FNE (Verma and Kaushik, 2017); FNE mediates the association between body image dissatisfaction and social anxiety, revealing the psychological chain from “insecurity about one's appearance” to “fear of being evaluated” (Levinson et al., 2013).

Fear of negative evaluation may further be associated with individuals' adoption of various behaviors intended to reduce the risk of being negatively evaluated. Specifically, to avoid negative evaluation due to appearance deficiencies, individuals high in FNE are more likely to use beauty filters to present a “safer” self-image to others (Lo Destro, 2024); to actively seek appearance-improvement information to substantively narrow the gap between their actual and ideal appearance; and to seek appearance feedback to confirm whether they are accepted and approved by others. Research indicates that FNE is positively associated with self-presentation behaviors and selfie focus (Bodroža et al., 2022); and a large-sample study of 2,657 participants found that low self-esteem was associated with stronger FNE, which in turn was associated with greater filter use (Lo Destro, 2024).

**H4:** FNE will show a positive indirect association linking appearance anxiety with all three appearance-oriented social media behaviors, such that higher appearance anxiety is associated with higher FNE, which in turn is associated with higher subsequent beauty-filter use, appearance information-seeking, and appearance feedback-seeking.

### 2.5 Moderating mechanism: media literacy

Media literacy refers to the capacity to identify, analyze, and critically evaluate media messages (Ren et al., 2026). In the context of social media appearance content, individuals high in media literacy can recognize that idealized appearances presented on platforms are predominantly filtered, edited, staged, and commercially packaged, and are thus less likely to regard such content as realistic or attainable standards (Alon et al., 2024). Theoretically, this critical cognition might attenuate the pathway linking appearance anxiety to behavior at two junctures: first, by recognizing that the appearance standards on social media are themselves distorted and unrealistic, individuals may reduce their fear of being evaluated for failing to meet these standards; second, it may weaken the translation of anxiety or FNE into specific behaviors, as critically aware individuals may be less prone to blindly pursuing distorted standards through photo editing, information-seeking, or feedback-seeking.

Existing research suggests that higher levels of media literacy (e.g., media skepticism, critical thinking about media messages) are associated with lower thin-ideal internalization, appearance comparison, and body dissatisfaction (Levinson and Rodebaugh, 2011), providing indirect support for the protective, buffering role media literacy may play (Buehler et al., 2026). Accordingly, the present study treats media literacy as a moderator, focusing on its moderation of the path from appearance anxiety to FNE as the core mediating pathway.

**H5:** Media literacy moderates the indirect association between appearance anxiety and appearance-oriented social media behaviors via FNE, such that the indirect pathway is attenuated at higher levels of media literacy.

Because the theoretical account above proposes that media literacy could attenuate the pathway at two junctures but H5 specifies moderation of only the first (the anxiety-to-FNE, or a-path, link), we treat moderation of the second (FNE-to-behavior, or b-path) link as an open, exploratory question rather than a pre-specified hypothesis, addressed in Section 4.6.

### 2.6 Bidirectional prospective associations

The present study further examines whether the predictive relationship between appearance anxiety and appearance-oriented social media behaviors is bidirectional over time. As reviewed in Section 2.1, emerging longitudinal evidence suggests that body image states can prospectively predict subsequent social media use behavior (Hawes et al., 2020). If the dynamic is truly cyclical, appearance-oriented social media behaviors should also positively predict subsequent appearance anxiety. Consistent with this expectation:

**H6:** Appearance anxiety and appearance-oriented social media behaviors will exhibit bidirectional prospective between-persons associations: appearance anxiety will positively predict subsequent appearance-oriented behaviors, and appearance-oriented behaviors will positively predict subsequent appearance anxiety.

In summary, extant research on social media and appearance anxiety has generated rich findings, but three lacunae remain: (1) excessive reliance on the unidirectional assumption of “social media use → appearance anxiety” at the level of causal direction, with systematic neglect of the reverse pathway; (2) methodological reliance on cross-sectional designs that cannot support inference about causal direction or dynamic change; and (3) failure to distinguish the specific behavioral strategies adopted by anxious individuals, with most studies measuring general use intensity rather than identifying distinct behavioral patterns such as beauty-filter use, appearance information-seeking, and appearance feedback-seeking.

**Figure 1:**
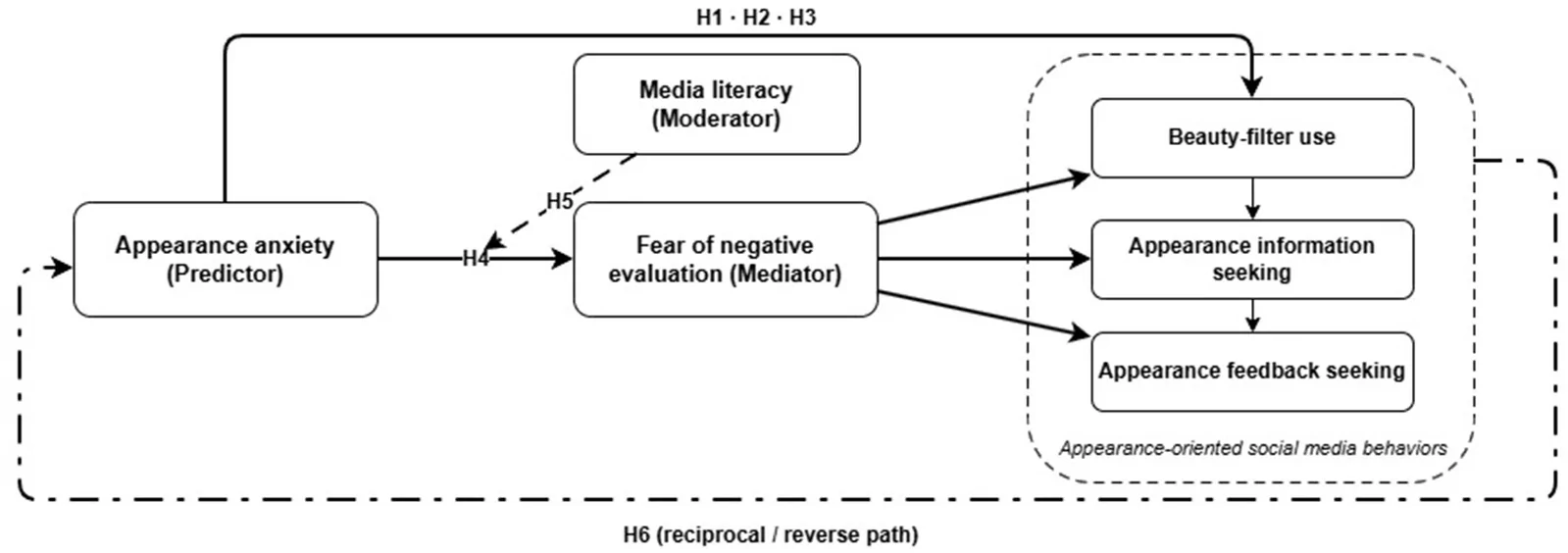
Mediating and moderating mechanisms linking appearance anxiety to appearance-oriented social media behaviors. Solid lines = direct predictive paths (H1–H4); dash-dot line = reciprocal/reverse path (H6); dashed line = moderating path (H5). The three behaviors were tested separately; for H6, the reverse path (behavior to anxiety) and the forward path held simultaneously.

To address these gaps, the present study employs UGT as its theoretical framework to construct a moderated, partially longitudinal indirect-association model: appearance anxiety as the independent variable, FNE as the candidate linking variable, three types of appearance-oriented social media behaviors as outcome variables, and media literacy as the moderator. A two-wave panel design with cross-lagged panel modeling is used to simultaneously examine paths in both directions. The proposed conceptual pathway is shown in Figure 1.

## 3 Method

### 3.1 Research design

This study employed a two-wave longitudinal panel design with young women as participants, examining appearance anxiety's prospective prediction of appearance-oriented social media behaviors, the candidate linking role of FNE, the moderating role of media literacy, and the bidirectional associations between appearance anxiety and appearance-oriented social media behaviors. The two measurement occasions (T1, T2) were separated by 6 months. The 6-month interval was chosen to balance “sufficient duration for observable change in behavioral habits” against “short enough to minimize sample attrition and interference from major life events”; this interval follows the precedent of prior longitudinal research in social media and body image (Lucas, 2023; Lüdtke and Trautwein, 2007).

The longitudinal design is the methodological centerpiece of this study. The vast majority of prior research on this topic has relied on cross-sectional designs that cannot support causal inference or capture within-person dynamic change. By using two-wave panel data and cross-lagged panel models (CLPMs), this study simultaneously estimates cross-lagged paths in both directions—“appearance anxiety → behavior” and “behavior → appearance anxiety”—enabling stronger inferences about the temporal ordering of these associations. It is important to note that two-wave CLPMs estimate between-persons prospective associations and cannot fully separate within-person change processes from stable between-person differences (Hamaker et al., 2015; Lucas, 2023). All cross-lagged estimates in this study should therefore be interpreted as between-persons prospective associations, not individual-level dynamic processes.

### 3.2 Participants and procedure

#### 3.2.1 Participants

Participants were young women from mainland China. Inclusion criteria were: (1) female by both biological sex and gender identity; (2) aged 18–35 years; (3) use of at least one of Weibo, Douyin, or Xiaohongshu for at least 3 days per week over the preceding 3 months. Exclusion criteria were: (1) questionnaire completion time substantially below threshold (< 5 min), indicating inattentive responding; (2) patterned responding (i.e., selecting the same option for all items); (3) duplicate submissions from the same IP address; and (4) failure to successfully match T1 data in the second wave.

Sample size was determined based on statistical power requirements for longitudinal indirect-effect models. Following power analysis recommendations for tests of indirect effects, with medium effect size, statistical power = 0.80, and α = 0.05, the minimum sample size for detecting indirect effects is approximately 150. Accounting for longitudinal attrition, T1 recruitment aimed for no fewer than 300 participants to ensure at least 200 successfully matched T2 participants, with acceptable attrition capped at 20%. The T1 wave yielded 412 returned questionnaires; after applying exclusion criteria, 389 were retained. Six months later, the T2 wave successfully matched and quality-screened 339 participants (retention rate: 87.1%), all of whom were included in final analyses.

To evaluate whether attrition between waves was associated with participants' baseline characteristics, the 339 participants retained at T2 were compared with the 50 T1 participants lost to follow-up on demographic variables and all T1 study variables; results are reported in Section 4.1.1.

#### 3.2.2 Procedure

Data were collected via a professional online survey platform, distributed through a combination of offline QR-code scanning at universities and targeted social media posting. Participants provided informed consent before proceeding. Participants created a privacy-protecting tracking code (last four digits of mobile number combined with birth month and day) to enable cross-wave data matching. Two reminders were sent during the 6-month interval (at months 2 and 4). A tiered incentive structure was employed: participants completing T1 received a small monetary reward; those completing both waves received a larger reward. Prior to formal data collection, a small-sample pilot study (*N* = 50) was conducted to assess questionnaire readability, average completion time, and preliminary scale reliability, and individual items that were unclear or had poor discriminability were revised accordingly. This study was approved by the Institutional Review Board of the authors' institution (approval number NO.NUST-SDAM-2025-113) and was conducted in accordance with the principles of the Declaration of Helsinki.

### 3.3 Measures

All measures were adapted from established domestic and international scales. For scales originally published in English, a standard translation–back-translation procedure was employed for Chinese adaptation, and content validity was assessed by experts in communication and psychology. Unless otherwise noted, all scales used five-point Likert scoring (1 = strongly disagree, 5 = strongly agree), with higher scores indicating higher levels of the relevant trait or behavior.

#### 3.3.1 Appearance anxiety

Appearance anxiety was measured using the Appearance Anxiety Scale (AAS), originally developed by Dion et al. (1990) and adapted into Chinese by Sun (2016). The scale comprises 14 items scored on a five-point Likert scale (1 = strongly disagree, 5 = strongly agree), with higher scores indicating higher appearance anxiety. In the present study, Cronbach's α = 0.954/0.943 and McDonald's ω = 0.955/0.944 at T1/T2, indicating excellent internal consistency.

#### 3.3.2 Fear of negative evaluation

FNE was measured using the Brief Fear of Negative Evaluation Scale–Straightforward items (BFNE-S; Rodebaugh et al., 2004), an eight-item unidimensional measure derived from Leary's (1983) original Brief Fear of Negative Evaluation Scale (BFNE). Items are scored on a five-point scale, with higher scores indicating greater fear of negative evaluation. In the present study, Cronbach's α = 0.909 and McDonald's ω = 0.909. Given that FNE represents a relatively stable dispositional trait, it was assessed only at baseline (T1) to minimize participant burden. Consequently, T1 FNE scores were used to evaluate a partially longitudinal indirect association within the broader prospective framework.

#### 3.3.3 Appearance-oriented social media behaviors

The present study distinguished three types of appearance-oriented social media behaviors with different gratification orientations, corresponding respectively to self-presentation management, information acquisition, and social validation needs. All three were assessed at both waves to examine change over time and bidirectional prediction.

Beauty-filter use was measured with a self-developed five-item scale assessing frequency and intensity of using beautification, smoothing, and slimming filters when publishing self-containing content on social media, and degree of dependence on filter functions (sample item: “I always use beauty or filter functions before posting selfies or photos of myself”). T1 Cronbach's α = 0.899.

Appearance information-seeking was measured with a self-developed five-item scale assessing frequency of actively searching for skincare, cosmetics, weight loss, and cosmetic-procedure content on social media, and following appearance-outstanding influencers (sample item: “I actively search for skincare, cosmetics, or weight-loss content on social media”). T1 Cronbach's α = 0.889.

Appearance feedback-seeking was measured with a self-developed four-item scale assessing attending to others' feedback (likes, comments) after posting appearance-related content, seeking appearance-related approval through carefully edited photos, and tendency to experience anxiety when feedback is insufficient (sample item: “I post content featuring my appearance primarily to receive likes and positive comments from others”). T1 Cronbach's α = 0.912.

The three scales were developed to operationalize the three gratification needs specified by uses-and-gratifications theory (self-presentation management, information acquisition, and social validation). Item pools were generated through a review of the Chinese- and English-language literature on appearance anxiety and social media use, supplemented by qualitative interviews. An initial version of each scale was then established through Delphi expert consultation and a pilot survey. Because the appropriate size of a Delphi panel depends on the disciplinary scope of the study, and the literature indicates an optimal range of 15–50 experts across 2–4 rounds of consultation, we selected 18 experts recognized for their authority, reliability, and disciplinary expertise to ensure professionally and scientifically grounded, multi-perspective input. Following the Delphi consultation, a pilot survey was conducted with 10 participants meeting the study's inclusion and exclusion criteria, informing the scales' finalization. During the pilot survey, two items originally included in the appearance feedback-seeking item pool were removed due to poor discriminant validity and redundancy with the scale's other items, yielding the final four-item measure; no items were removed from the beauty-filter use or appearance information-seeking pools.

As with the appearance anxiety and FNE items, participants rated each behavior item on the same five-point agreement scale (1 = strongly disagree, 5 = strongly agree) with reference to their actual situation over the past month (see the instructions preceding these items in Supplementary Appendix S1A); the scales therefore capture subjective agreement with descriptive statements about one's own behavior over a defined recall period, rather than counts of behavioral frequency. Full Chinese items and English translations for all scales, together with standardized factor loadings and item residual variances, are provided in Supplementary Appendix S1A. Confirmatory factor analysis (CFA) was conducted in the formal analysis to further examine their factor structures (results in Section 4.2), including a comparison against alternative (multi-factor) structures given that the beauty-filter scale spans frequency, intensity, and dependence facets, and the feedback-seeking scale includes an item referencing negative affect when feedback is insufficient (Section 4.2); a sensitivity analysis excluding this item is reported in Section 4.2 as well. We acknowledge that, as self-developed measures, the psychometric properties of these scales beyond internal consistency remain to be established in independent samples; convergent validity against established behavioral measures should be a priority in future research.

#### 3.3.4 Media literacy

Media literacy was measured using a Chinese adaptation of the Critical Thinking about Media Messages–Appearance Focused subscale (CTMM-AF), developed by McLean et al. (2016) on the basis of Scull et al. (2010), adapted for the context of social media appearance content. The scale comprises six items assessing individuals' capacity to identify, analyze, and critically evaluate modified or idealized appearance content on social media (sample item: “I know that appearance images on social media have mostly been digitally altered and do not represent real appearances”). Given that media literacy is expected to be relatively stable, this variable was assessed only at T1. In the present study, Cronbach's α = 0.830 and McDonald's ω = 0.832.

#### 3.3.5 Control variables

Demographic and social media use information collected at T1 included age, education level, self-reported body mass index (BMI), daily social media use duration, and primary platform used. These variables were considered as potential controls, but the reported main analyses used the pre-specified demographic covariate set of age, education, and BMI. Daily social media use duration and primary platform are reported in the attrition and descriptive analyses, but were not included in the main CLPM or indirect-association models because the focal outcomes are themselves appearance-oriented social media behaviors; adjusting for broad social media use or platform preference could remove substantive variance in the behaviors under study and would make the covariate set less comparable across models. At T2, two additional items screened participants for major life events during the 6-month interval that might have affected their appearance-related concerns or social media use; these items were used for screening and contextual checks rather than as covariates in the reported main analyses.

To reduce participant burden and T2 attrition, the T2 questionnaire reassessed only core variables (appearance anxiety, three appearance-oriented behaviors) and did not repeat FNE, media literacy, or demographic items; total item count was capped at 40 items with an estimated completion time of 8–10 min. Item wording was identical across waves for reassessed variables.

### 3.4 Data analysis

Data were analyzed using IBM SPSS Statistics (IBM Corp., Armonk, NY, USA) and Mplus 8.3 (Muthén & Muthén, Los Angeles, CA, USA) in three steps.

Missing data were addressed at two levels. First, unit-level wave non-response was defined as valid T1 completion without a matched, quality-screened T2 questionnaire. Fifty of 389 valid T1 respondents (12.9%) met this definition and were excluded from the longitudinal analytic sample; their baseline characteristics were compared with those of retained participants in the attrition analysis reported in Section 4.1.1. Second, item-level missingness was checked after screening and matching. The final analysis file contained complete substantive item responses for all retained cases on the T1 and T2 variables used in the reported models (0 missing item values across the analyzed scale items). Scale scores were therefore calculated as the arithmetic mean of the corresponding items, and no item-level imputation was performed. Because the matched analytic dataset was complete for the variables included in the CLPM and mediation/moderation analyses, models were estimated on the matched complete-case sample (*N* = 339) using MLR in Mplus.

Step 1: preliminary analyses, including: (1) common method bias assessment via Harman's single-factor test (supplemented by interpretive caution given known limitations of this method); (2) descriptive statistics and correlations; (3) reliability (Cronbach's α, McDonald's ω) and validity assessment (CFA, AVE, CR, and HTMT), with model fit evaluated against CFI and TLI ≥ 0.90, RMSEA and SRMR ≤ 0.08 (Hu and Bentler, 1999); and (4) longitudinal measurement invariance testing to confirm equivalence of factor loadings and intercepts across T1 and T2.

Step 2: cross-lagged panel analysis (testing H1–H3 and H6). To examine the prospective between-persons predictive direction between appearance anxiety and appearance-oriented behaviors, CLPMs were constructed that simultaneously incorporated appearance anxiety and each appearance behavior at both waves. Controlling for baseline levels, age, education, and BMI, two cross-lagged paths were estimated for each model: prediction of T2 behavior from T1 appearance anxiety (H1–H3), and prediction of T2 appearance anxiety from T1 behavior (H6). Separate CLPMs, rather than a single multivariate model incorporating all three behaviors simultaneously, were estimated for each behavior for two reasons. First, H1–H3 and H6 concern the prospective association between appearance anxiety and each behavior individually, not their joint or relative prediction, so a multivariate specification was not required to test them. Second, although the three behaviors are conceptually and empirically distinct (Section 2.2; see the CFA comparison in Section 4.2), they are moderately correlated at T1 (rs = 0.41–0.48; Table 2); modeling all three simultaneously as either predictors or outcomes of appearance anxiety would introduce shared-variance overlap among the behavior variables that could alter each behavior-specific path in ways that would complicate interpretation relative to the behavior-specific hypotheses under test. We note as a limitation, however, that because the three behaviors were not modeled jointly, the present estimates do not indicate whether anxiety's prospective association with one behavior is partly redundant with its association with the other two; a multivariate specification modeling all three behaviors and their covariances simultaneously would be a natural extension for future research seeking to partition their unique and shared prediction. All models were estimated with robust maximum likelihood (MLR).

**Table 2:** Descriptive statistics and intercorrelation matrix (T1 and T2).

Variable	*M* (SD)	1	2	3	4	5	6	7	8	9
1. Appearance anxiety (T1)	3.38 (1.00)	—								
2. FNE (T1)	3.37 (1.07)	0.47^***^	—							
3. Beauty-filter use (T1)	3.26 (1.20)	0.46^***^	0.49^***^	—						
4. Appearance information-seeking (T1)	3.33 (1.23)	0.48^***^	0.34^***^	0.41^***^	—					
5. Appearance feedback-seeking (T1)	3.37 (1.22)	0.47^***^	0.52^***^	0.48^***^	0.43^***^	—				
6. Media literacy (T1)	2.93 (1.08)	−0.44^***^	−0.41^***^	−0.47^***^	−0.22^***^	−0.41^***^	—			
7. Appearance anxiety (T2)	3.10 (1.16)	0.47^***^	0.52^***^	0.57^***^	0.52^***^	0.62^***^	−0.29^***^	—		
8.Beauty-filter use (T2)	3.18 (1.18)	0.49^***^	0.45^***^	0.58^***^	0.43^***^	0.51^***^	−0.39^***^	0.48^***^	—	
9. Appearance information-seeking (T2)	3.33 (1.14)	0.53^***^	0.46^***^	0.39^***^	0.47^***^	0.48^***^	−0.21^***^	0.43^***^	0.30^***^	—
10. Appearance feedback-seeking (T2)	3.49 (1.11)	0.46^***^	0.50^***^	0.48^***^	0.43^***^	0.48^***^	−0.21^***^	0.51^***^	0.35^***^	0.37^***^

*N* = 339. FNE, fear of negative evaluation. Media literacy was assessed only at T1. Column numbers correspond to the numbered variables in the first column (variable 10 is omitted as a column because it has no further correlations to display).

^***^p < 0.001.

Because only two waves were available, a formally identified RI-CLPM was not estimated. Instead, we conducted an assumption-dependent phantom-variable sensitivity analysis. For each two-construct model, observed standardized scores for appearance anxiety (X1, X2) and one appearance behavior (Y1, Y2) were decomposed into fixed stable phantom components (RI_X and RI_Y) and occasion-specific components (wX1, wX2, wY1, and wY2). The stable phantom factors loaded 1.00 on both waves of their respective constructs; occasion-specific factors loaded 1.00 on their corresponding observed indicators; and manifest residual variances were fixed to 0 so that each observed score was represented as the sum of its stable and occasion-specific components. Stable-factor means were fixed to 0, stable factors were constrained to be orthogonal to all occasion-specific factors, and the within-wave covariances wX1 WITH wY1 and wX2 WITH wY2 were estimated. The autoregressive and cross-lagged paths wX2 ON wX1 wY1 and wY2 ON wY1 wX1 were estimated, with age, education, and BMI retained as covariates. Identification required externally imposed stable-component values. In the primary specification, Var(RI_X) and Var(RI_Y) were fixed to 0.40 in standardized units. The stable-factor covariance was fixed as kappa x rXY_T1, where kappa is the assumed stable variance share and rXY_T1 is the observed T1 correlation between appearance anxiety and the focal behavior from Table 2 (0.46 for beauty-filter use, 0.48 for appearance information-seeking, and 0.47 for appearance feedback-seeking). Thus, the primary fixed stable covariances were 0.184, 0.192, and 0.188, respectively. The 0.40 primary value was selected because same-construct 6-month stability correlations were moderate (0.47–0.58; Table 2), making 0.40 a plausible but not maximal stable-share assumption. To evaluate robustness, the models were re-run with kappa fixed at 0.25 and 0.55, yielding fixed stable covariances of 0.115/0.120/0.118 and 0.253/0.264/0.259 for beauty-filter use, information-seeking, and feedback-seeking, respectively. All other fixed parameters and estimated paths were identical across the grid. Complete Mplus syntax for the primary and alternative specifications is provided in Supplementary Appendix S1B.

Step 3: moderated indirect-association analysis (testing H4 and H5). To examine a candidate linking pathway between appearance anxiety and behavior and its boundary conditions, a moderated indirect-association model was constructed with T1 appearance anxiety as the independent variable, T1 FNE as the candidate linking variable, T1 media literacy as the moderator, and T2 behavior for each of the three behavior types as the outcome, additionally controlling for age, education, BMI, and the corresponding T1 (baseline) level of each behavior. Indirect effects and the index of moderated indirect association were estimated using bias-corrected bootstrapping (5,000 samples); indirect effects were considered significant if their 95% CIs excluded zero; the moderated indirect-association index was considered significant if its 95% CI excluded zero. As an exploratory extension addressing the possibility that media literacy also moderates the FNE-to-behavior (b-path) link, not only the anxiety-to-FNE (a-path) link specified in H5, we additionally estimated a model in which media literacy moderated both paths simultaneously, with conditional indirect effects computed at the mean and ±1 SD of media literacy for each behavior (results in Section 4.6 and Table 3). Because this extension was not part of the pre-specified hypothesis set, it is interpreted as exploratory.

**Table 3:** Exploratory test of media literacy moderating the FNE-to-behavior (b-path) link.

Behavior	FNE × ML interaction *b* (SE)	*p-*value	Moderated indirect-association index (95% CI)	Indirect effect at −1 SD ML	Indirect effect at mean ML	Indirect effect at +1 SD ML
Beauty-filter use	0.133 (0.039)	0.001	0.049 (0.021, 0.090)	−0.012 (−0.076, 0.049)	0.043 (−0.012, 0.100)^a^	0.080 (0.031, 0.157)
Appearance information-seeking	0.036 (0.038)	0.342	0.013 (−0.013, 0.043)	0.102 (0.036, 0.188)	0.101 (0.053, 0.165)	0.096 (0.043, 0.171)
Appearance feedback-seeking	0.021 (0.036)	0.555	0.008 (−0.017, 0.035)	0.110 (0.047, 0.194)	0.103 (0.056, 0.167)	0.092 (0.044, 0.164)

*N* = 339. ML, media literacy (grand-mean centered). Model additionally includes media literacy moderation of the a-path (anxiety → FNE), age, education, BMI, and each behavior's T1 (baseline) level (not shown); see Table 9 for a-path results, which are unchanged from the model without *b*-path moderation. Indirect and moderated-mediation index estimates use bias-corrected bootstrap 95% CIs (5,000 samples); bracketed CIs excluding zero are significant at *p* < 0.05. This is an exploratory, post hoc extension (Section 3.4) not part of the pre-specified hypothesis set; interpret with caution pending replication.

^a^The mean-ML conditional indirect effect for beauty-filter use is not statistically significant by either method: p = 0.082 by the delta-method Wald test, and its bias-corrected bootstrap 95% CI (−0.012, 0.100) also includes zero; we report both given the known non-normality of indirect-effect sampling distributions.

We did not apply a formal correction (e.g., Bonferroni or false discovery rate) for multiple comparisons across the full set of analyses (six CLPM cross-lagged paths, three indirect-association models, and the moderation and exploratory b-path moderation models). This reflects a deliberate trade-off: the primary CLPM and indirect-association analyses tested a small number of pre-specified, theoretically motivated hypotheses (H1–H6) rather than an exploratory search across many possible associations, for which correction is most clearly warranted, and a stringent family-wise correction across all hypothesis tests in the study would substantially reduce power for each individual pre-specified test. We made an explicit exception for the exploratory b-path moderation extension (Section 4.6), which was not pre-specified; there, we flagged the absence of correction directly and noted that only one of three tests reached significance, treating that result as a hypothesis for confirmatory replication rather than an established effect (Section 5.4). Readers should nonetheless bear the absence of correction in mind when weighing the full set of reported significant findings, particularly for results outside the pre-specified hypothesis set.

After the corrected analyses were completed, the authors conducted a numerical consistency audit. All results affected by the corrections, including sample-size and retention figures, descriptive statistics, reliability and validity indices, correlations, CLPM coefficients and fit indices, mediation and moderation estimates, figure annotations, and Supplementary material S1 sensitivity results, were checked against the final analysis outputs across the Abstract, main text, tables, figures, and Supplementary material S1. Reported values were independently checked by two members of the research team, with discrepancies resolved against the analysis logs before finalization.

## 4 Results

### 4.1 Preliminary data screening

#### 4.1.1 Missing data and attrition analysis

To assess whether attrition between T1 and T2 introduced systematic bias, the 339 participants retained at T2 were compared with the 50 T1 participants who were not retained on all baseline demographic variables (age, education level, BMI, daily social media use duration, primary platform) and T1 study variables (appearance anxiety, FNE, media literacy, and the three appearance-oriented behaviors), using independent-samples *t*-tests for continuous variables and χ^2^ tests for categorical variables. Results are presented in Table 4.

**Table 4:** Comparison of baseline (T1) characteristics between participants retained at T2 and those lost to follow-up.

Variable	Retained at T2 (*n* = 339)	Lost to follow-up (*n* = 50)	Test statistic	*p-*value
Age (years)	26.53 (5.03)	29.14 (6.46)	*t*_(387)_ = −3.30	0.001
Education level	1: 35 (10.3%); 2: 86 (25.4%); 3: 171 (50.4%); 4: 47 (13.9%)	1: 5 (10.0%); 2: 13 (26.0%); 3: 23 (46.0%); 4: 9 (18.0%)	$\chi_{\left(3\right)}^{2}$ = 0.70	0.873
BMI	21.63 (2.74)	21.65 (2.49)	*t*_(387)_ = −0.05	0.959
Daily social media use duration (1–5)	3.05 (1.04)	3.10 (0.95)	*t*_(387)_ = −0.32	0.750
Primary platform	1: 64 (18.9%); 2: 112 (33.0%); 3: 144 (42.5%); 4: 19 (5.6%)	1: 8 (16.0%); 2: 24 (48.0%); 3: 18 (36.0%); 4: 0 (0.0%)	$\chi_{\left(3\right)}^{2}$ = 6.23	0.101
Appearance anxiety (T1)	3.38 (1.00)	3.08 (0.93)	*t*_(387)_ = 2.01	0.046
Fear of negative evaluation (T1)	3.37 (1.07)	3.15 (0.98)	*t*_(387)_ = 1.37	0.173
Media literacy (T1)	2.93 (1.08)	3.37 (1.01)	*t*_(387)_ = −2.74	0.006
Beauty-filter use (T1)	3.26 (1.20)	3.30 (0.99)	*t*_(387)_ = −0.24	0.813
Appearance information-seeking (T1)	3.33 (1.23)	3.11 (0.99)	*t*_(387)_ = 1.21	0.229
Appearance feedback-seeking (T1)	3.37 (1.22)	3.13 (1.10)	*t*_(387)_ = 1.30	0.196

Retained *n* = 339; Lost to Follow-Up *n* = 50 (of *N* = 389 T1 completers). Continuous variables were compared using independent-samples t-tests; categorical variables were compared using chi-square tests of independence. Daily social media use duration is reported using the original 1–5 questionnaire code. Education level and primary platform percentages are shown by questionnaire code.

Retained participants were significantly younger than those lost to follow-up (*M* = 26.53, SD = 5.03 vs. *M* = 29.14, SD = 6.46), *t*_(387)_ = −3.30, *p* = 0.001. They also reported higher T1 appearance anxiety (*M* = 3.38, SD = 1.00 vs. *M* = 3.08, SD = 0.93), *t*_(387)_ = 2.01, *p* = 0.046, and lower T1 media literacy (*M* = 2.93, SD = 1.08 vs. *M* = 3.37, SD = 1.01), *t*_(387)_ = −2.74, *p* = 0.006. No significant differences were observed for education level, BMI, daily social media use duration, primary platform, T1 FNE, T1 beauty-filter use, T1 appearance information-seeking, or T1 appearance feedback-seeking (*p*s ≥ 0.101). Thus, although the retention rate was high, attrition was not completely random with respect to all baseline characteristics; longitudinal findings should be interpreted with this attrition pattern in mind.

#### 4.1.2 Common method bias

As all data derive from participant self-report, Harman's single-factor test was used as an initial check for common method bias. The unrotated first factor accounted for 36.7% of variance, below the conventional 40% threshold. However, Harman's single-factor test cannot rule out common method variance, and a result below this threshold should not be interpreted as evidence that common method bias is not severe; the test is, at best, a weak diagnostic. The correlational pattern of results should accordingly be considered in light of potential inflation due to common method variance, and future research should employ stronger design-based or statistical controls (e.g., marker-variable technique, temporal or source separation of measures) to address this limitation more rigorously. Associations estimated within the same wave (e.g., the anxiety-FNE a-path in the indirect-association model, and the T1 correlations underlying Table 2) are more susceptible to this inflation, since anxiety, FNE, and T1 behaviors were all self-reported concurrently. The prospective cross-lagged and indirect paths, which link constructs measured 6 months apart, are comparatively less susceptible to same-occasion common method effects, though they are not immune, since both waves still rely on the same reporters and response format.

### 4.2 Reliability, validity, and measurement invariance

Reliability and validity results for each scale are presented in Table 5. Cronbach's alpha coefficients ranged from 0.830 to 0.954 and McDonald's omega coefficients ranged from 0.832 to 0.955 across scales, all exceeding the 0.70 threshold. CFA-based CR exceeded 0.70 for all scales; AVE exceeded 0.50 for all scales except Media Literacy (AVE = 0.458), although the AVE for Media Literacy was slightly below the conventional 0.50 criterion, its CR exceeded 0.70, providing additional evidence of adequate internal consistency (Fornell and Larcker, 1981). HTMT ratios between constructs ranged from 0.25 to 0.57, all below the 0.85 threshold, indicating adequate discriminant validity. Because appearance anxiety was assessed at both waves and used in the cross-lagged models, its T2 reliability and validity coefficients are now reported in Table 5.

**Table 5:** Reliability and convergent validity of scales.

Variable	Items	*α* (T1/T2)	*ω* (T1/T2)	AVE (T1/T2)	CR (T1/T2)
Appearance anxiety	14	0.954/0.943	0.955/0.944	0.601/0.549	0.955/0.944
Fear of negative evaluation	8	0.909/—	0.909/—	0.555/—	0.909/—
Beauty-filter use	5	0.899/0.903	0.903/0.907	0.652/0.663	0.903/0.907
Appearance information-seeking	5	0.889/0.891	0.891/0.899	0.622/0.642	0.891/0.899
Appearance feedback-seeking	4	0.912/0.881	0.912/0.882	0.721/0.651	0.912/0.882
Media LITERACY	6	0.830/—	0.832/—	0.458/—	0.832/—

*N* = 339. α,Cronbach's α; ω, McDonald's ω; AVE, average variance extracted; CR, composite reliability. Variables assessed only at T1 are indicated with “—” for T2. All HTMT values (0.25–0.57) < 0.85. Appearance-anxiety T2 coefficients were calculated from the matched T2 item responses.

Longitudinal measurement invariance was evaluated separately for each construct assessed at both waves (appearance anxiety, beauty-filter use, appearance information-seeking, and appearance feedback-seeking). The model sequence comprised configural invariance (same factor structure across T1 and T2), metric invariance (factor loadings constrained equal across waves), and scalar invariance (factor loadings and item intercepts constrained equal across waves). The configural model served as the reference model; therefore, ΔCFI and ΔRMSEA are reported only for the metric-vs.-configural and scalar-vs.-metric comparisons. Complete fit indices for each successive model are reported in Table 6. Across constructs, successive changes in fit generally supported longitudinal invariance: all ΔCFI values were ≤ 0.010 except for the appearance-anxiety scalar comparison, which was marginally above the criterion after rounding (ΔCFI = −0.011), and all ΔRMSEA values were ≤ 0.015 except for the beauty-filter metric comparison, which was marginally above the criterion after rounding (ΔRMSEA = 0.016). Scalar invariance was therefore judged acceptable overall, with these two borderline deviations noted. Scalar invariance is interpreted as support for both invariant factor loadings and invariant item intercepts across waves, thereby supporting cross-wave comparisons of latent means and longitudinal paths.

**Table 6:** Longitudinal measurement invariance for constructs assessed at both waves.

Construct	Model	χ^2^	df	CFI	TLI	RMSEA	SRMR	ΔCFI	ΔRMSEA
AA	Configural	673.07	335	0.949	0.943	0.055	0.036	–	–
AA	Metric	716.47	348	0.945	0.940	0.056	0.054	−0.005	0.001
AA	Scalar	801.68	361	0.934	0.931	0.060	0.055	−0.011	0.004
BF	Configural	26.00	29	1.000	1.002	0.000	0.017	–	–
BF	Metric	35.88	33	0.999	0.998	0.016	0.033	−0.001	0.016
BF	Scalar	41.17	37	0.998	0.998	0.018	0.033	−0.001	0.002
IS	Configural	78.67	29	0.976	0.962	0.071	0.031	–	–
IS	Metric	80.70	33	0.977	0.968	0.065	0.033	0.001	−0.006
IS	Scalar	90.35	37	0.974	0.968	0.065	0.033	−0.003	0.000
FS	Configural	38.06	15	0.987	0.975	0.067	0.030	–	–
FS	Metric	44.01	18	0.985	0.977	0.065	0.039	−0.002	−0.002
FS	Scalar	60.50	21	0.977	0.970	0.075	0.039	−0.008	0.009

AA, appearance anxiety; BF, beauty-filter use; IS, appearance information-seeking; FS, appearance feedback-seeking.

The configural model is the reference model, so ΔCFI and ΔRMSEA are not reported for that row. Metric Δ values compare the metric model with the configural model; scalar Δ values compare the scalar model with the metric model. Fit indices are from longitudinal CFA models estimated on the matched two-wave item responses with correlated uniquenesses for matched items. Common decision rules are ΔCFI < = 0.010 and ΔRMSEA < = 0.015 for supporting the more constrained model.

Standardized factor loadings and item residual variances for all scales are reported in Supplementary Appendix Table A1. Loadings ranged from 0.48 to 0.92; the great majority exceeded 0.70, with five exceptions (A14 = 0.69, B1 = 0.64, M2 = 0.65, M5 = 0.61, M6 = 0.48), three of which belong to the Media Literacy scale, consistent with that scale's comparatively lower AVE (Table 5). Single-factor models fit the data well for each self-developed behavior scale individually (beauty-filter: CFI = 1.00, RMSEA = 0.014; information-seeking: CFI = 0.99, RMSEA = 0.067; feedback-seeking: CFI = 0.99, RMSEA = 0.125, though this estimate is based on only 2 degrees of freedom and should be interpreted cautiously). Because the beauty-filter scale's items span frequency, intensity, and dependence facets of filter use, and the feedback-seeking scale includes an item referencing negative affect, we additionally examined the eigenvalue structure of each scale's inter-item correlation matrix as a check on unidimensionality. For all three scales, only the first eigenvalue exceeded 1 (beauty-filter: 3.60, 0.47, 0.36, 0.29, 0.28; information-seeking: 3.48, 0.48, 0.43, 0.35, 0.26; feedback-seeking: 3.16, 0.35, 0.30, 0.18), a pattern consistent with a single dominant factor and providing no support for a multi-factor alternative structure for any of the three scales.

Because the feedback-seeking scale includes one item referencing negative affect when feedback is insufficient (“If my appearance-related posts receive little feedback, I feel anxious or dejected”; C34 in Supplementary Appendix S1A), which may conceptually overlap with appearance anxiety and FNE, we conducted a sensitivity analysis re-estimating the key CLPM and indirect-association paths for feedback-seeking with this item excluded (3-item composite). Using simplified OLS models with the same covariates as the primary analyses (for computational tractability; official estimates use the full Mplus specification), results were materially unchanged: the path from T1 appearance anxiety to T2 feedback-seeking remained significant (*β* = 0.281 vs. 0.306 for the full scale), as did the indirect-association model's b-path (FNE *β* = 0.251 vs. 0.241) and direct effect (anxiety *β* = 0.204 vs.0.229). A parallel check dropping the information-seeking item referencing appearance dissatisfaction (“When I feel dissatisfied with my appearance, I browse beauty-related content more frequently”; C23) likewise left results essentially unchanged (CLPM *β* = 0.400 vs. 0.395; b-path = 0.211 vs. 0.223; direct effect = 0.306 vs. 0.298). These checks suggest that the observed associations are not primarily driven by item-level content overlap between the behavior scales and appearance anxiety or FNE, though we did not re-run the full bias-corrected bootstrap procedure with these items excluded.

### 4.3 Descriptive statistics and correlations

Descriptive statistics and correlations are presented in Table 2, which now reports both T1 and T2 variables together with their cross-wave correlations. Appearance anxiety was significantly positively correlated with all three appearance-oriented behaviors (rs = 0.46–0.48, *p*s < 0.001) and with FNE (*r* = 0.47, *p* < 0.001); media literacy was significantly negatively correlated with all appearance behaviors and with FNE (rs = −0.21 to −0.47, *p*s < 0.001). Same-construct T1–T2 stability correlations were moderate to moderately high (appearance anxiety *r* = 0.47; beauty-filter use *r* = 0.58; appearance information-seeking *r* = 0.47; appearance feedback-seeking *r* = 0.48; all *p*s < 0.001), and cross-wave, cross-construct correlations followed a broadly similar pattern to the T1 correlations reported above (e.g., T1 anxiety with T2 behaviors, rs = 0.46–0.53). Paired-samples *t*-tests indicated that appearance anxiety was significantly higher at baseline (T1: *M* = 3.38, SD = 1.00) than 6 months later (T2: *M* = 3.10, SD = 1.16), *t*_(338)_ = 4.51, *p* < 0.001, Cohen's *d* = 0.24 (using the SD of difference scores), suggesting a modest overall decline in anxiety over the follow-up period, while the relative rank ordering of individuals remained moderately stable (*r* = 0.47, *p* < 0.001).

### 4.4 Cross-lagged panel analysis: bidirectional between-persons associations

**Figure 2:**
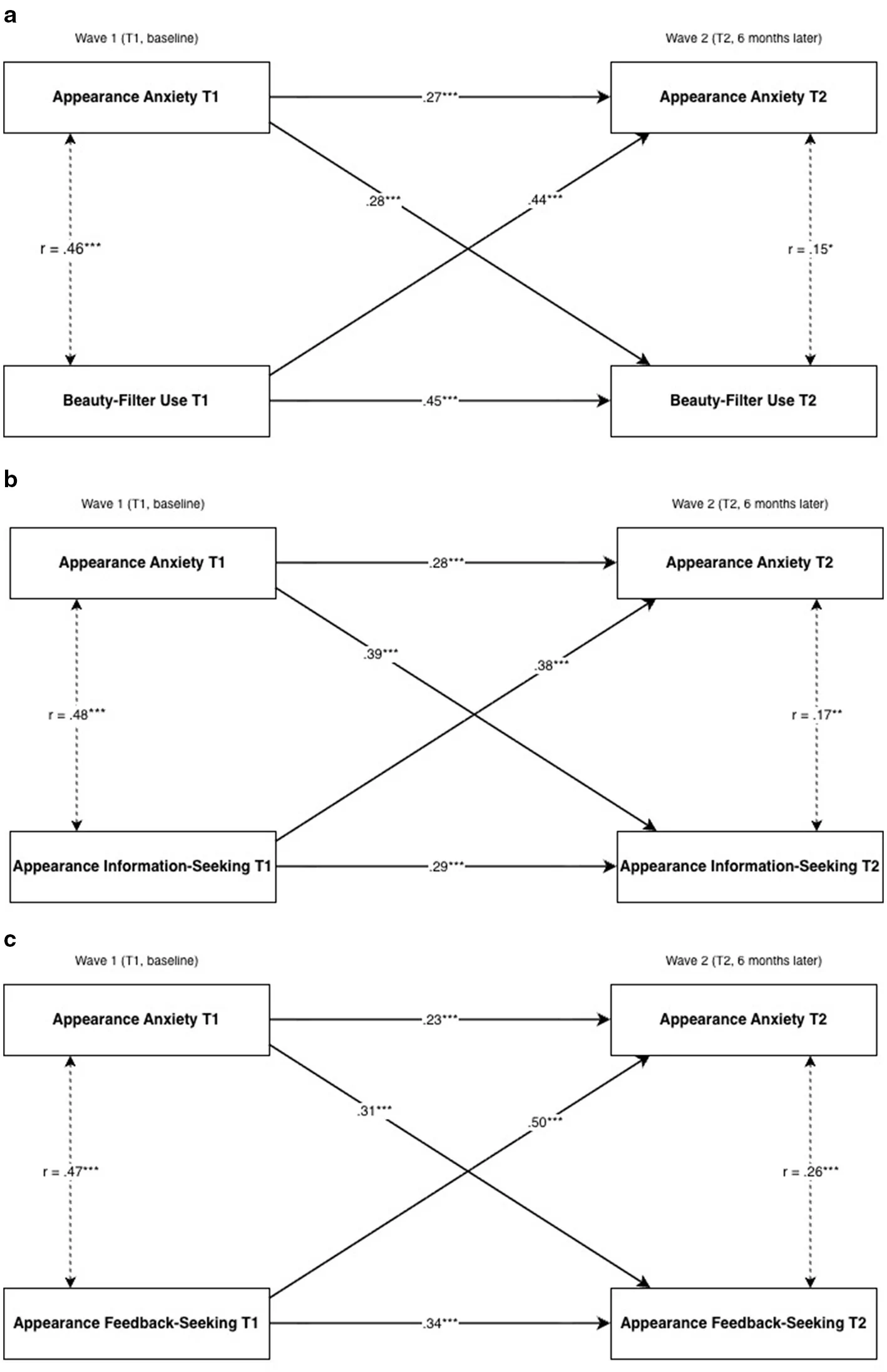
**(a)** Cross-lagged panel model: appearance anxiety and beauty-filter use. **(b)** Cross-lagged panel model: appearance anxiety and appearance information-seeking. **(c)** Cross-lagged panel model: appearance anxiety and appearance feedback-seeking. Path line thickness is uniform and does not encode effect size. *N* = 339. Standardized coefficients are shown; all paths are drawn with uniform line weight. Solid arrows denote directional paths (controlling for baseline levels, age, education, and BMI); dashed double-headed arrows denote correlations. *p < 0.05, **p < 0.01, ***p < 0.001.

CLPMs constructed separately for each of the three appearance-oriented behaviors all demonstrated good model fit (CFI ≥ 0.985, TLI ≥ 0.972, RMSEA ≤ 0.046, SRMR ≤ 0.041). Results are presented in Table 7. The corresponding cross-lagged diagrams are shown in Figure 2.

**Table 7:** Cross-lagged panel model path coefficients (standardized *β* and 95% CI).

Behavior type	Hypothesis	Anxiety T1 → Behavior T2 [*β* (95% CI)]	Behavior T1 → Anxiety T2 [*β* (95% CI)]	Model fit TLI/CFI
Beauty-filter use	H1	+0.280^***^ (0.189, 0.371)	+0.446^***^ (0.339, 0.553)	0.985/1.000
Appearance information-seeking	H2	+0.394^***^ (0.297, 0.491)	+0.377^***^ (0.270, 0.484)	1.000/1.000
Appearance feedback-seeking	H3	+0.305^***^ (0.210, 0.400)	+0.499^***^ (0.399, 0.599)	0.991/0.995

*N* = 339, MLR estimation.

^***^p < 0.001.

All models control for baseline, age, education, and BMI. H6 is supported by all three reverse-direction paths.

Controlling for baseline levels and demographic variables, T1 appearance anxiety significantly and positively predicted T2 beauty-filter use [*β* = 0.280, 95% CI (0.189, 0.371), *p* < 0.001], T2 appearance information-seeking [*β* = 0.394, 95% CI (0.297, 0.491), *p* < 0.001], and T2 appearance feedback-seeking [*β* = 0.305, 95% CI (0.210, 0.400), *p* < 0.001] at the between-persons level, supporting H1, H2, and H3. Simultaneously, the reverse paths were also significant: T1 beauty-filter use [*β* = 0.446, 95% CI (0.339, 0.553), *p* < 0.001], T1 appearance information-seeking [*β* = 0.377, 95% CI (0.270, 0.484), *p* < 0.001], and T1 appearance feedback-seeking [*β* = 0.499, 95% CI (0.399, 0.599), *p* < 0.001] each significantly and positively predicted T2 appearance anxiety at the between-persons level, supporting H6.

These bidirectional between-persons associations were further examined in the assumption-dependent phantom-variable sensitivity analysis described in Section 3.4 and Supplementary Appendix S1B. Across the primary specification (kappa = 0.40) and the alternative fixed stable-variance assumptions (kappa = 0.25 and 0.55), the forward and reverse cross-lagged paths retained the same positive direction and remained statistically significant for all three behavior models. However, the magnitude of the paths varied across fixed assumptions, confirming that this analysis is assumption-dependent. Accordingly, the sensitivity analysis is interpreted only as evidence that the qualitative bidirectional pattern is not eliminated under the tested fixed stable-component assumptions; it is not treated as a formally identified RI-CLPM, as a full removal of stable between-person differences, or as evidence of within-person reciprocal processes.

Comparing the magnitude of path coefficients in the two directions is descriptively informative but should be interpreted cautiously: because paths are estimated in separate equations with differing residual structures, formal comparisons would require Wald tests or model constraints, which were not the primary focus of this study. Descriptively, the reverse paths (T1 behaviors → T2 anxiety, *β*s = 0.38–0.50) tended to be of comparable or larger magnitude than the forward paths (T1 anxiety → T2 behaviors, *β*s = 0.28–0.39), consistent with the possibility that appearance-oriented behaviors are associated with elevated subsequent anxiety at the between-persons level.

### 4.5 Indirect association analysis: fear of negative evaluation as candidate link

Indirect-association results are presented in Table 8. Each model predicts T2 behavior from T1 appearance anxiety and T1 FNE, additionally controlling for age, education, BMI, and the corresponding T1 (baseline) level of that behavior. Appearance anxiety was significantly and positively associated with FNE (a-path, *β* = 0.370, *p* < 0.001); FNE in turn significantly and positively predicted all three T2 appearance behaviors after this baseline-behavior control (b-paths, *β*s = 0.15–0.26, *p*s < 0.05). Bias-corrected bootstrapping (5,000 samples) indicated significant indirect associations through FNE for all three behaviors after controlling for baseline behavior: beauty-filter use [indirect effect = 0.057, 95% CI (0.016, 0.119)]; appearance information-seeking [indirect effect = 0.092, 95% CI (0.046, 0.155)]; and appearance feedback-seeking [indirect effect = 0.095, 95% CI (0.049, 0.159)]. All three 95% CIs excluded zero, supporting H4, though the beauty-filter indirect association was smaller and closer to zero than the other two. After controlling for FNE and baseline behavior, the direct associations of appearance anxiety with all three behaviors remained significant and were larger than the corresponding indirect associations (*β*s =0.235–0.339, *p*s < 0.001), indicating that FNE accounts for a modest share of anxiety's total prospective association with subsequent behavior.

**Table 8:** Indirect associations through fear of negative evaluation, controlling for baseline behavior (bootstrap, 5,000 samples).

Indirect path	a-Path *β*	b-Path *β*	Indirect effect	95% CI	Direct effect *β*
Anxiety → FNE → Beauty-filter use	0.370^***^	0.153^*^	0.057	(0.016, 0.119)	0.258^***^
Anxiety → FNE → Appearance information-seeking	0.370^***^	0.249^***^	0.092	(0.046, 0.155)	0.339^***^
Anxiety → FNE → Appearance feedback-seeking	0.370^***^	0.257^***^	0.095	(0.049, 0.159)	0.235^***^

*N* = 339. FNE, fear of negative evaluation. Each model additionally controls for age, education, BMI, and the corresponding T1 (baseline) level of the outcome behavior (not shown). All indirect effects have 95% CIs excluding zero. Because appearance anxiety and FNE were measured at the same wave, these estimates are interpreted as indirect associations rather than evidence of a causal mediation process.

^*^p < 0.05; ^***^p < 0.001.

As a robustness check on the direction of this pathway, we additionally estimated an alternative-order model in which appearance anxiety and FNE exchanged roles (T1 FNE → T1 appearance anxiety → T2 behavior; see Section 5.3 for interpretation). The reverse first-stage association (FNE → anxiety, *β* = 0.414, *p* < 0.001) was, if anything, numerically larger than the hypothesized a-path (anxiety → FNE, *β* = 0.370), and the resulting indirect effects [beauty-filter use = 0.107, 95% CI (0.056, 0.171); information-seeking = 0.140, 95% CI (0.089, 0.207); feedback-seeking = 0.097, 95% CI (0.051, 0.158)] were of comparable or larger magnitude than those from the hypothesized model. Because appearance anxiety and FNE were measured at the same wave, the present data cannot statistically adjudicate between these two directions.

### 4.6 Moderation analysis: media literacy as moderator

**Figure 3:**
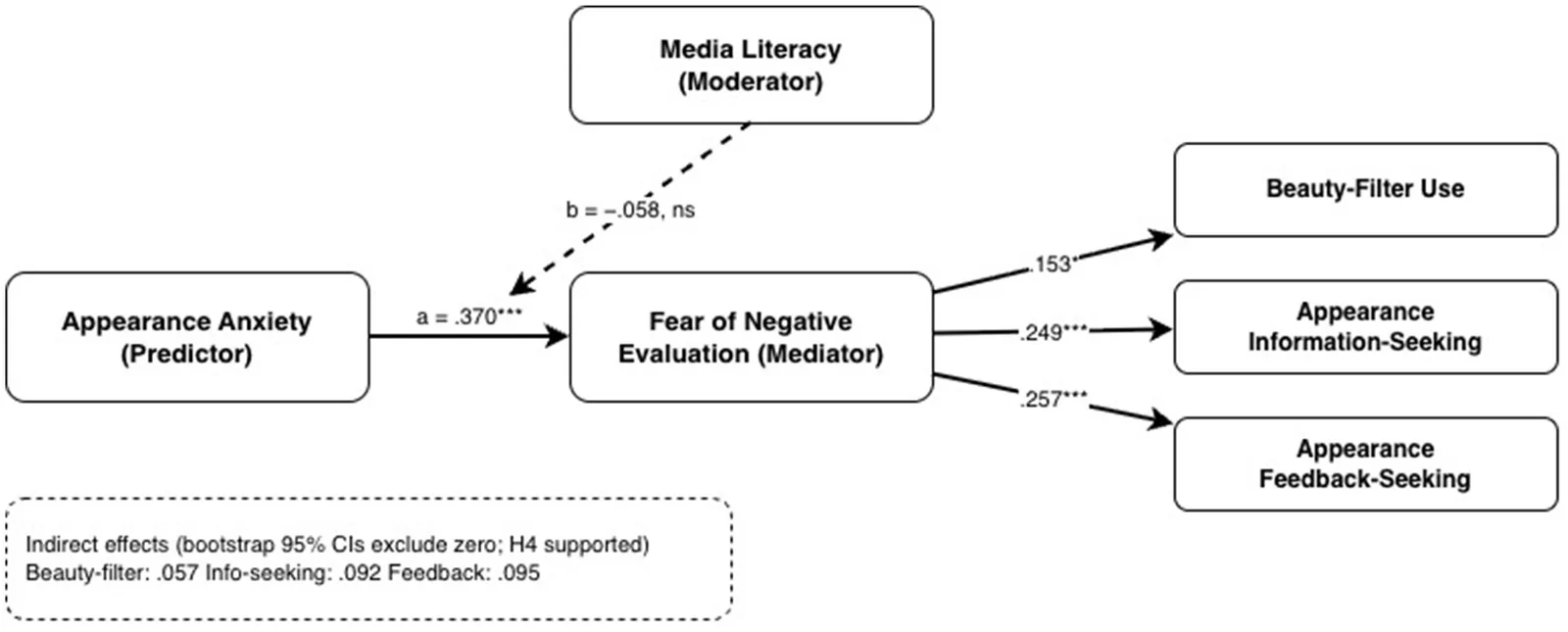
Moderated indirect-association model results. a = anxiety → fear of negative evaluation; b-paths are listed for beauty-filter/Information-seeking/ feedback-seeking in order. The dashed line is the (non-significant) moderating path. Direct effects (c') are reported in Table 2. *p < 0.05, ***p < 0.001.

Moderation analysis results are presented in Table 9, and the model is shown in Figure 3. Prior to computing the product term, T1 appearance anxiety was standardized (*M* = 0, SD = 1) and T1 media literacy was mean-centered (not scaled); the interaction term was the product of these two centered variables, and moderation was modeled on the a-path (appearance anxiety → FNE). The interaction term of appearance anxiety × media literacy did not significantly predict FNE [unstandardized *b* = −0.058, SE = 0.043, 95% CI (−0.141, 0.027), *p* = 0.174]. The moderated indirect-association indices computed separately for each of the three behaviors all had 95% CIs including zero (see Table 9). These results indicate that the present study did not find statistically reliable evidence that media literacy attenuates the indirect association between appearance anxiety and appearance behaviors via FNE, and H5 was not supported; given the modest *post-hoc* power for this analysis (≈62%; see Section 5.4), a small moderating effect cannot be ruled out.

**Table 9:** Moderation effect of media literacy and moderated indirect-association indices.

Interaction term	*b* (unstd.)	SE	95% CI	*p-*value	Moderated indirect-association index 95% CI
Anxiety × Media literacy → FNE → Beauty-filter use	−0.058	0.043	(−0.141, 0.027)	0.174	(−0.030, 0.002) (includes 0)
Anxiety × Media literacy → FNE → Appearance information-seeking	−0.058	0.043	(−0.141, 0.027)	0.174	(−0.040, 0.005) (includes 0)
Anxiety × Media literacy → FNE → Appearance feedback-seeking	−0.058	0.043	(−0.141, 0.027)	0.174	(−0.042, 0.005) (includes 0)

*N* = 339. The interaction term (appearance anxiety × media literacy) operates on the a-path ( → FNE). Prior to computing the product term, appearance anxiety was standardized (M = 0, SD = 1) and media literacy was mean-centered (not scaled). *b*, unstandardized coefficient. All moderated indirect-association indices have 95% CIs including zero; the present study did not find statistically reliable evidence for H5, though a small effect cannot be ruled out given the limited power of this analysis (post-hoc power ≈ 62%).

As an exploratory extension, we additionally tested whether media literacy moderates the FNE-to-behavior (b-path) link, which the theoretical model (Section 2.5) also proposed but H5 did not formally specify. Results are presented in Table 3. The FNE × media literacy interaction significantly predicted T2 beauty-filter use [*b* = 0.133, SE = 0.039, 95% CI (0.057, 0.210), *p* = 0.001] but not T2 appearance information-seeking (*b* = 0.036, *p* = 0.342) or T2 appearance feedback-seeking (*b* = 0.021, *p* = 0.555). The corresponding moderated indirect-association index was significant for beauty-filter use [0.049, 95% CI (0.021, 0.090)] but not for the other two behaviors. Contrary to the direction proposed in Section 2.5, the conditional indirect effect of anxiety on beauty-filter use via FNE was near zero and non-significant among individuals with low media literacy [−1 SD: −0.012, 95% CI (−0.076, 0.049)], but significant and substantially larger among individuals with high media literacy [+1 SD: 0.080, 95% CI (0.031, 0.157)]. For appearance information-seeking and appearance feedback-seeking, the indirect effect via FNE remained significant and similar in magnitude across low, mean, and high levels of media literacy (Table 3), consistent with the absence of significant *b*-path moderation for these two behaviors. Given the exploratory, *post hoc* nature of this test across three behaviors, this finding should be interpreted cautiously pending replication.

### 4.7 Interpreting the magnitude of effects

To move beyond statistical significance, the standardized coefficients reported above can be interpreted most cautiously in relation to one another and to the design of the present study, rather than by directly applying benchmarks developed for zero-order correlation coefficients. The forward cross-lagged paths from appearance anxiety to later appearance-oriented behaviors (*β*s =0.28–0.39) and the reverse-direction paths from behavior to later anxiety (*β*s =0.38–0.50; Table 7) were consistently positive and statistically reliable, with the reverse-direction associations generally comparable to or larger than the forward associations. These values suggest that the associations are not trivial within this sample and model specification, but they should not be categorized as “medium” or “large” effects without additional justification for applying correlation-based thresholds to standardized cross-lagged regression coefficients (Funder and Ozer, 2019). By contrast, the FNE indirect associations (0.057–0.095; Table 8) were smaller in absolute magnitude than the corresponding direct associations involving anxiety (0.235–0.339), indicating that FNE is best understood as one modest explanatory contributor among others (Section 5.5) rather than the primary pathway linking anxiety to behavior. The non-significant a-path moderation by media literacy (Table 9), and the single significant but exploratory b-path effect for beauty-filter use (Table 3), should both be weighed against this study's limited power to detect interaction effects and should not be treated as precise estimates of the true population effect.

## 5 Discussion

### 5.1 Appearance anxiety as a prospective between-persons predictor of appearance-oriented behaviors

The present study found significant positive prospective between-persons associations between T1 appearance anxiety and T2 beauty-filter use, appearance information-seeking, and appearance feedback-seeking. This result is consistent with the core argument derived from UGT: appearance anxiety, as a persistent state of negative self-evaluation, may function as an internal psychological need state associated with later specific media use behaviors. At the between-persons level, higher appearance anxiety was associated with higher levels of beauty-filter use, appearance information-seeking, and appearance feedback-seeking—a pattern consistent with the possibility that these behaviors function, respectively, as strategies for managing online self-presentation, acquiring improvement information, and obtaining external validation, though the present between-persons design cannot confirm that any given individual engages in these behaviors for these specific motivational reasons. This finding is consistent with longitudinal evidence in prior research suggesting that body image states prospectively predict subsequent social media use behavior (Hawes et al., 2020), and extends that logic to the level of differentiated, active appearance-oriented behaviors, responding to UGT's core assertion about the motivating force of “gratifications sought” (Palmgreen and Rayburn, 1982).

### 5.2 Bidirectional between-persons reinforcement of appearance anxiety and appearance behaviors

The most important finding of the present study is evidence of bidirectional between-persons prospective associations between appearance anxiety and appearance-oriented behaviors: higher anxiety was associated with higher subsequent behavior, and higher behavior was likewise associated with higher subsequent anxiety. At the between-persons level, individuals who were higher in appearance-oriented social media behaviors at T1 also tended to be higher in appearance anxiety at T2. This suggests that appearance-oriented behaviors—including repeatedly modifying self-images, sustained exposure to idealized appearance content, and continually seeking appearance feedback—are associated with greater subsequent appearance concern.

These bidirectional between-persons associations remained in the same direction and were statistically significant in the assumption-dependent phantom-variable sensitivity analysis, but this result should be interpreted cautiously because the two-wave phantom model depends on externally fixed stable-component variances and covariances. We therefore use the analysis only as a robustness check of the qualitative sign and significance of the cross-lagged paths. It should not be interpreted as establishing within-person dynamic cycles or as fully removing stable between-person differences. Future research employing three or more waves with a formally identified RI-CLPM would provide a stronger test of within-person dynamics.

These findings offer a complement and challenge to the dominant unidirectional paradigm of “social media use → appearance anxiety” in the literature, and are consistent with recent longitudinal research emphasizing reciprocal influences between body image and social media behavior (Demetriou et al., 2025). We reiterate, however, that these are between-persons associations open to alternative accounts: stable individual differences such as trait negative affectivity, perfectionism, or general psychological distress could contribute to both sides of this association without either construct causally influencing the other (Section 2.3; see also Section 5.5). From a practical standpoint, and with the caveat that an observational design cannot directly establish that intervening on one variable would change the other, these findings tentatively suggest that interventions targeting either appearance anxiety or appearance-oriented behaviors in isolation may be insufficient; both may warrant simultaneous attention, a possibility that would need to be tested in dedicated intervention research rather than inferred from the present design alone.

### 5.3 The explanatory role of fear of negative evaluation

The present study found significant positive indirect associations through FNE between appearance anxiety and all three appearance behaviors, suggesting a potential explanatory pathway by which anxiety may be associated with subsequent behavior. Individuals higher in appearance anxiety may lack confidence in how others will evaluate their appearance, rendering them more susceptible to fear of negative evaluation; to reduce the risk of being negatively evaluated, they may turn to social media behaviors that manage their appearance image, acquire improvement methods, or confirm their attractiveness. This indirect-association pathway contributes to understanding of how anxiety may be associated with subsequent behavior, and is consistent with existing research showing that FNE serves as a bridge between body image dissatisfaction and social behavior (Levinson et al., 2013).

It is noteworthy that the direct associations of appearance anxiety with all three behaviors remained significant after controlling for FNE and baseline behavior, and were consistently larger than the corresponding FNE indirect associations (direct *β*s = 0.235–0.339 vs. indirect *β*s = 0.057–0.095). This pattern indicates that FNE accounts for only a modest share of anxiety's total prospective association with subsequent behavior. Appearance anxiety may also be associated with appearance-oriented behaviors through other mechanisms (e.g., direct emotional reactivity, habituated behavioral patterns), and future research can further explore other potential linking variables.

Several important limitations of the indirect-association analysis should be acknowledged. Because appearance anxiety and FNE were both assessed at T1, the model is more accurately described as a partially longitudinal indirect-association model: the appearance anxiety-FNE relation (a-path) is cross-sectional, while the FNE-behavior relation (b-path) is prospective (T1 FNE → T2 behavior). Because appearance anxiety and FNE were measured simultaneously, their relative temporal order cannot be established, and the analysis cannot rule out that FNE precedes appearance anxiety or that the two constructs partly overlap conceptually—a concern reinforced by the fact that appearance anxiety is itself defined partly in terms of concern about others' evaluations. Accordingly, statements that FNE “linked” the association should be read as shorthand for findings that are consistent with an indirect association through FNE, rather than as evidence for the proposed causal direction. As a direct test of this concern, we additionally estimated an alternative-order model in which appearance anxiety and FNE exchanged roles (T1 FNE → T1 appearance anxiety → T2 behavior). The reverse first-stage association (FNE → anxiety, *β* = 0.414, *p* < 0.001) was, if anything, numerically larger than the hypothesized a-path (anxiety → FNE, *β* = 0.370), and the resulting indirect effects (beauty-filter use = 0.107; information-seeking = 0.140; feedback-seeking = 0.097; all 95% CIs excluding zero) were of comparable or larger magnitude than those from the hypothesized model (0.057–0.095). Because appearance anxiety and FNE were measured at the same wave, the present data cannot statistically adjudicate between these two directions on this basis alone; the anxiety-to-FNE ordering favored in the main analysis rests on theoretical grounds (UGT's emphasis on anxiety as an antecedent need state) rather than on evidence that this direction fits the data better than the reverse. Future research should include FNE measurement at multiple waves (e.g., T1, T2, T3 in a three-wave design) to construct a fully longitudinal indirect model (e.g., T1 anxiety → T2 FNE → T3 behaviors) capable of establishing temporal precedence.

### 5.4 The non-significant moderation effect of media literacy

Contrary to expectations, the present study did not find statistically reliable evidence that cognitive media literacy moderated the proposed indirect association [interaction *b* = −0.058, SE = 0.043, 95% CI (−0.141, 0.027), *p* = 0.174], and H5 was not supported. A non-significant interaction indicates that this study did not find reliable evidence of moderation; it does not establish the absence of a protective effect. This is especially relevant given the modest *post-hoc* power for this analysis (≈62%), so a small moderating effect cannot be ruled out. This result warrants consideration from both theoretical and methodological perspectives.

From a theoretical standpoint, one plausible explanation is that the type of media literacy assessed in this study—critical cognition regarding the modified nature of social media appearance content—may be insufficient to override the emotional and evaluative processes underlying the association between appearance anxiety and behavior. According to the knowledge-behavior gap literature, individuals may fail to translate critical awareness into behavioral change when emotional arousal is high (Livingstone and Helsper, 2006). When individuals are in a state of heightened appearance anxiety and fear of negative evaluation, rational cognitive awareness of media distortion may be unable to effectively interrupt the emotionally driven process. This interpretation is consistent with dual-process theories, which predict that System 2 reasoning is suppressed in contexts dominated by System 1 affective processing.

From a methodological standpoint, the media literacy scale used primarily captured the cognitive dimension of “recognizing that appearance content is modified,” without covering the behavioral activation dimension of media literacy (i.e., the capacity to translate critical cognition into active resistance). Future research could consider adopting more comprehensive media literacy measures to more fully examine its moderating potential.

The exploratory extension testing b-path moderation (Section 4.6, Table 3) produced a more nuanced picture than the null a-path result above. Media literacy significantly moderated the FNE-to-behavior link for beauty-filter use, but in the opposite direction from that proposed in Section 2.5: rather than weakening the association between FNE and filter use, higher media literacy was associated with a stronger indirect path from appearance anxiety through FNE to beauty-filter use, while this indirect effect was near zero and non-significant among individuals with low media literacy. No significant b-path moderation emerged for information-seeking or feedback-seeking, where the indirect effect via FNE remained fairly stable across levels of media literacy.

This reversed pattern for beauty-filter use, though unexpected, admits a plausible reading: recognizing how pervasively appearance content is edited may not reduce one's own motivation to edit when anxious, and could instead sharpen the perceived cost of appearing comparatively unfiltered. Filter use is also the one of the three behaviors that directly and immediately controls one's own image, unlike information-seeking or feedback-seeking, which may make it more sensitive to this kind of normative reasoning. A more methodologically cautious reading is also warranted: three b-path interactions were tested without correction for multiple comparisons, and only one reached significance, so this finding should be treated as a hypothesis for confirmatory replication rather than an established effect.

These tentative findings, if replicated in adequately powered samples, would tentatively suggest that interventions targeting appearance anxiety in young women may need to go beyond general media literacy education, more directly addressing emotional regulation and negative self-evaluation. The exploratory b-path finding for beauty-filter use adds a further caution: if higher media literacy is indeed associated with a stronger, not weaker, link between FNE and filter use, then media literacy education alone should not be assumed to be protective for this behavior, and may warrant closer evaluation before being adopted as an intervention component. For example, cognitive restructuring techniques from cognitive-behavioral therapy or mindfulness practices aimed at reducing emotional reactivity to appearance evaluation may be worth exploring as complementary approaches. The present study did not test or compare any interventions, however, and cannot speak to whether media-literacy, cognitive-behavioral, or mindfulness-based approaches would be more effective than one another; this would require a dedicated comparative intervention trial. Given that the present null result also cannot rule out a small moderating effect, this recommendation should be treated as provisional pending replication.

### 5.5 Limitations and future directions

Several limitations of this study should be acknowledged.

(1) The two-wave cross-lagged design enables inferences about temporal ordering of between-persons associations, but two-wave CLPMs are mathematically equivalent to partial difference score models and cannot fully separate within-person change processes from stable between-person differences (Hamaker et al., 2015; Lucas, 2023). All cross-lagged estimates should accordingly be interpreted as between-persons prospective associations. Future research employing three or more waves and RI-CLPM would enable purer estimation of within-person dynamic processes.
(2) The FNE and media literacy were assessed only at baseline. Because appearance anxiety and FNE were measured simultaneously, the indirect-association and moderation analyses are best described as partially longitudinal (the anxiety-FNE path is cross-sectional; the FNE-behavior path is prospective), and the relative temporal order of appearance anxiety and FNE cannot be established from the present data. A robustness check reversing the hypothesized ordering (FNE → appearance anxiety → behavior; see Section 5.3) produced indirect effects of comparable or larger magnitude than the hypothesized model, confirming that the present two-wave design cannot statistically distinguish between these directions. Future research should incorporate FNE into longitudinal measurement via a three-wave design (T1 anxiety → T2 FNE → T3 behaviors) to support stronger causal inference about the indirect pathway.
(3) The three appearance-oriented behavior scales are self-developed. Although reliability and CFA-based structural evidence was established in this sample, independent convergent validity evidence against established behavioral measures is needed before these scales can be widely adopted. Useful next steps would include correlating scale scores with passively logged behavioral data and co-administering the scales alongside existing related measures in an independent sample to more directly establish convergent and external validity.
(4) The study sample consisted of young Chinese women recruited primarily through universities and social media platforms, resulting in limited representativeness and diversity. Moreover, the recruitment method may have introduced selection bias: individuals more engaged with social media. The Chinese social media platform ecology (Xiaohongshu, Douyin, etc.) differs substantially from Western platforms; findings should be generalized to other cultural contexts with caution. Future research should include more diverse samples (different cultural backgrounds, genders, broader age ranges) to examine the generalizability of the findings. Additionally, although the assumption-dependent phantom-variable sensitivity analysis provided partial reassurance regarding the robustness of the bidirectional associations under the tested fixed stable-component assumptions, a formally specified RI-CLPM with three or more waves remains the definitive next step for this research program. Although retention was high (87.1%), the attrition analysis indicated that participants lost to follow-up were older, reported lower T1 appearance anxiety, and reported higher T1 media literacy than those retained, so conclusions based on T2 data should be generalized with caution.
(5) The models did not control for psychological variables known to influence both appearance anxiety and social media behavior, such as depressive symptoms, self-esteem, perfectionism, disordered eating symptoms, or general (non-appearance-specific) social anxiety. Although the present hypotheses are grounded in appearance-specific theoretical mechanisms (Section 2.3) rather than general negative affectivity, and the CLPM models control for baseline levels of the outcome alongside age, education, and BMI, we cannot rule out that some portion of the observed prospective associations reflects shared variance with these unmeasured constructs rather than a process specific to appearance anxiety (see also the third-variable and general-engagement alternative accounts discussed in Section 2.3). Future research should incorporate these variables as covariates or comparison predictors to isolate the unique contribution of appearance anxiety.

In addition, all variables, including the three appearance-oriented behaviors, were evaluated via self-reports instead of objective recordings. Self-reported behavioral measures are prone to recall errors and social expectation biases, which is especially likely in this context as appearance management behaviors such as using filters or seeking feedback may be regarded as indicative of insecurity. If participants underestimated these behaviors or their association with anxiety due to considerations of social desirability, then the true associations may be underestimated, and other forms of self-report bias may overstate these associations. Future research that combines self-reports with passively recorded behavioral data, when available, will assist in determining whether the current associations are applicable to objective measures rather than merely self-perceived behavior.

## 6 Conclusion

Based on a two-wave panel design in the Chinese platform ecology of Xiaohongshu, Douyin, and Weibo, this study provides evidence of prospective between-persons associations between appearance anxiety and all three types of appearance-oriented social media behaviors (beauty-filter use, appearance information-seeking, and appearance feedback-seeking), with reverse-direction associations of comparable or larger magnitude. These bidirectional between-persons associations were retained in an assumption-dependent phantom-variable sensitivity analysis, but that analysis is not formally equivalent to an identified RI-CLPM and does not establish within-person reciprocal processes. The findings were consistent with FNE functioning as one candidate dispositional link between appearance anxiety and later appearance-management behaviors, based on a partially longitudinal indirect-association model that controlled for baseline behavior and showed significant, though modest, indirect associations across all three behavioral outcomes alongside larger direct associations involving anxiety; a robustness check reversing the ordering of appearance anxiety and FNE produced indirect associations of comparable or larger magnitude, underscoring that this design cannot establish which construct precedes the other. Cognitive-level media literacy did not moderate the anxiety-to-FNE pathway as hypothesized; an exploratory test found it did significantly moderate the FNE-to-behavior link for beauty-filter use, but in the opposite direction from that theorized, a finding that awaits confirmatory replication. Taken together, these findings should be understood as evidence for prospective, between-persons longitudinal associations between appearance anxiety and appearance-oriented social media behaviors, not as demonstration of a definitive reciprocal causal process; the present two-wave design, reliance on self-report measures, and unmeasured psychological confounds (Section 5.5) all counsel this more limited interpretation. Future research should prioritize three-wave RI-CLPM replication with T2 FNE measurement, passive behavioral measurement, and probability sampling to advance causal inference in this domain.

## Data Availability

The original contributions presented in the study are included in the article/Supplementary material, further inquiries can be directed to the corresponding author.
