# Supplementary material for: Appearance anxiety and appearance-management behaviors on Chinese social media: evidence for prospective bidirectional associations
Source: Front Psychol. 2026 Sep 3;17:1926306. doi: 10.3389/fpsyg.2026.1926306 (PMC13582449; doi:10.3389/fpsyg.2026.1926306)
Supplement: Supplementary file 1 [file Supplementary_File_1.docx]

# **Appendix A**

Full items for all scales are provided below in their original Chinese with English translations, together with standardized factor loadings (T1) and item residual variances from the confirmatory factor analyses reported in Section 4.2. All items used a five-point agreement scale (1 = strongly disagree/very inconsistent [很不符合], 5 = strongly agree/very consistent [很符合]), administered with the instruction to respond based on one's actual situation over the past month. Items so noted were reverse-scored prior to computing scale composites. The appearance anxiety, fear of negative evaluation, and media literacy scales are Chinese adaptations of previously published instruments (citations given in Section 3.3); the beauty-filter use, appearance information-seeking, and appearance feedback-seeking scales were developed for this study.

**Table A1**

*Full Item Wording, English Translations, and Standardized Factor Loadings*

| **Code** | **Chinese Item (中文条目)** | **English Translation** | **Loading** | **Residual** |
| --- | --- | --- | --- | --- |
| ***Appearance Anxiety (AAS; Dion et al., 1990; adapted by Sun, 2016)*** | | | | |
| A1 | 我会对自己的外表感到紧张不安。 | I feel nervous and anxious about my appearance. | 0.745 | 0.444 |
| A2 | 我担心别人如何评价我的相貌。 | I worry about how others evaluate my looks. | 0.755 | 0.430 |
| A3 | 我很满意自己的外表。 | I am satisfied with my appearance. (reverse-scored) | 0.727 | 0.472 |
| A4 | 我喜欢我的样子。 | I like the way I look. (reverse-scored) | 0.772 | 0.404 |
| A5 | 我想改变我的样子。 | I want to change the way I look. | 0.815 | 0.335 |
| A6 | 我对自己的体形或身材感到满意。 | I am satisfied with my body shape or figure. (reverse-scored) | 0.744 | 0.446 |
| A7 | 我对自己外表的某些方面感觉不舒服。 | I feel uncomfortable about certain aspects of my appearance. | 0.778 | 0.395 |
| A8 | 我觉得我的大多数朋友的外表都比我更迷人。 | I think most of my friends are more attractive than I am. | 0.804 | 0.354 |
| A9 | 我希望自己更好看。 | I wish I were better looking. | 0.857 | 0.265 |
| A10 | 我担忧自己吸引伴侣的能力。 | I worry about my ability to attract a partner. | 0.748 | 0.441 |
| A11 | 我对自己的长相感到舒服。 | I feel comfortable about the way I look. (reverse-scored) | 0.830 | 0.310 |
| A12 | 我很满意自己的体重。 | I am satisfied with my weight. (reverse-scored) | 0.838 | 0.297 |
| A13 | 当别人评论我的外表时，我会很紧张激动。 | When others comment on my appearance, I become very nervous and agitated. | 0.727 | 0.471 |
| A14 | 我很自信别人认为我很有吸引力。 | I am confident that others find me attractive. (reverse-scored) | 0.692 | 0.521 |
| ***Fear of Negative Evaluation (BFNE-S; Rodebaugh et al., 2004)*** | | | | |
| B1 | 即使我知道别人的看法无关紧要，我还是担心别人会怎样看我。 | I worry about what people think of me even when I know it doesn't make a difference. | 0.643 | 0.586 |
| B2 | 我经常害怕别人指出我的缺点。 | I am frequently afraid of other people noticing my shortcomings. | 0.789 | 0.377 |
| B3 | 我害怕得不到别人的认可。 | I am afraid others will not approve of me. | 0.756 | 0.428 |
| B4 | 我害怕别人会挑我的毛病。 | I am afraid that people will find fault with me. | 0.735 | 0.459 |
| B5 | 当我跟别人谈话时，我担心他们会怎样看我。 | When I am talking to someone, I worry about what they may be thinking of me. | 0.713 | 0.492 |
| B6 | 我时常担心我给别人留下了什么样的印象。 | I am usually worried about what kind of impression I make. | 0.745 | 0.444 |
| B7 | 有时我觉得自己过于担心别人怎样看我。 | Sometimes I think I am too concerned with what other people think of me. | 0.789 | 0.378 |
| B8 | 我通常担心我会说错话或做错事。 | I often worry that I will say or do the wrong things. | 0.778 | 0.395 |
| ***Beauty-Filter Use (self-developed)*** | | | | |
| C11 | 发布含有我自己的照片或视频时，我会使用美颜、磨皮、瘦脸等滤镜功能。 | When posting photos or videos of myself, I use beautification, smoothing, or face-slimming filter functions. | 0.830 | 0.311 |
| C12 | 我会在发布前反复调整滤镜或修图效果，直到对自己的外貌满意为止。 | I repeatedly adjust filters or photo-editing effects before posting until I am satisfied with my appearance. | 0.713 | 0.491 |
| C13 | 使用美颜功能后，我会更愿意在社交媒体上发布自己的照片。 | After using beautification functions, I am more willing to post photos of myself on social media. | 0.846 | 0.284 |
| C14 | 如果无法使用美颜滤镜，我会倾向于不发布含有自己面部的内容。 | If I cannot use beauty filters, I tend not to post content showing my face. | 0.851 | 0.277 |
| C15 | 我会主动尝试和探索新的美颜、修图或外貌调整功能。 | I actively try out and explore new beautification, photo-editing, or appearance-adjustment features. | 0.790 | 0.375 |
| ***Appearance Information-Seeking (self-developed)*** | | | | |
| C21 | 我会主动在社交媒体上搜索护肤、美妆、穿搭等外貌相关内容。 | I actively search social media for skincare, makeup, fashion, and other appearance-related content. | 0.726 | 0.473 |
| C22 | 我会关注外貌出众的博主或网红，浏览他们展示外貌的内容。 | I follow bloggers or influencers with outstanding appearances and browse their appearance-related content. | 0.745 | 0.446 |
| C23 | 当我对自己的外貌感到不满意时，会更频繁地浏览与美貌相关的内容。 | When I feel dissatisfied with my appearance, I browse beauty-related content more frequently. | 0.803 | 0.356 |
| C24 | 我会主动搜索医美、整形、瘦身等方面的信息。 | I actively search for information on cosmetic procedures, plastic surgery, or weight loss. | 0.791 | 0.374 |
| C25 | 我会花时间研究改善自己外貌的方法和技巧。 | I spend time researching methods and techniques to improve my appearance. | 0.870 | 0.243 |
| ***Appearance Feedback-Seeking (self-developed)*** | | | | |
| C31 | 发布自拍或含有我外貌的内容后，我会频繁查看点赞和评论。 | After posting selfies or appearance-related content, I frequently check likes and comments. | 0.811 | 0.343 |
| C32 | 他人对我外貌的正面评价（如"好看""变美了"）会让我感到明显的满足。 | Positive comments about my appearance from others (e.g., "you look good," "you've gotten prettier") make me feel clearly satisfied. | 0.868 | 0.247 |
| C33 | 我会通过发布精心修饰过的照片，来确认自己是否有吸引力。 | I post carefully edited photos to confirm whether I am attractive. | 0.798 | 0.362 |
| C34 | 如果我发布的外貌相关内容获得的反馈很少，我会感到焦虑或失落。 | If my appearance-related posts receive little feedback, I feel anxious or dejected. | 0.915 | 0.162 |
| ***Media Literacy (CTMM-AF; McLean et al., 2016, based on Scull et al., 2010)*** | | | | |
| M1 | 当我看到社交媒体上展示理想外貌的内容时，我会思考这条内容背后的目的是什么。 | When I see content showcasing an ideal appearance on social media, I think about what purpose is behind it. | 0.776 | 0.398 |
| M2 | 当我看到外貌出众的博主或网红发布外貌内容时，我会思考这条内容是谁制作或发布的。 | When I see appearance content posted by attractive bloggers or influencers, I think about who produced or posted it. | 0.646 | 0.582 |
| M3 | 当我浏览展示完美外貌的社交媒体内容时，我会思考发布者想让我相信什么。 | When I browse social media content showing perfect appearances, I think about what the poster wants me to believe. | 0.762 | 0.419 |
| M4 | 当我看到外貌出众的博主发布的内容时，我会思考对方用了哪些手法（如滤镜、修图、摆拍、广告植入）来吸引我的注意。 | When I see content posted by attractive bloggers, I think about what techniques (e.g., filters, editing, posing, product placement) they used to catch my attention. | 0.736 | 0.459 |
| M5 | 当我看到鼓励变美的社交媒体内容时，我会思考发布者希望我做的事（如购买产品、模仿某种外貌）是否真的对我有益。 | When I see social media content that encourages beautification, I think about whether what the poster wants me to do (e.g., buy a product, imitate a certain look) is truly good for me. | 0.613 | 0.624 |
| M6 | 当我看到展示理想外貌或身材的内容时，我会思考它在多大程度上是真实的，还是被美化或虚构的。 | When I see content showing an ideal appearance or figure, I think about the extent to which it is real versus beautified or fabricated. | 0.482 | 0.768 |
