# Supplementary material for: Appearance anxiety and appearance-management behaviors on Chinese social media: evidence for prospective bidirectional associations
Source: Front Psychol. 2026 Sep 3;17:1926306. doi: 10.3389/fpsyg.2026.1926306 (PMC13582449; doi:10.3389/fpsyg.2026.1926306)
Supplement: Supplementary file 2 [file Supplementary_File_2.docx]

# **Appendix B**

# Supplementary Appendix: Mplus Syntax and Key Output for Revised Analyses

This appendix provides the reproducibility materials requested for the revised analyses. The accompanying ZIP folder contains complete Mplus input files for the corrected CLPM, partially longitudinal mediation, moderation, exploratory moderated-mediation, and assumption-dependent phantom-variable sensitivity analyses. Key output values used to verify the revised manuscript are summarized below.

Data basis. The analytic panel includes the retained matched sample (N = 339). The expected composite-score data file for the CLPM, mediation, and moderation syntax is appearance_panel_analysis.dat, with the following variable order: id age education bmi AA_T1 FNE_T1 ML_T1 BF_T1 IS_T1 FS_T1 AA_T2 BF_T2 IS_T2 FS_T2. The missing-value code is -999.

**Table S1. Syntax file index**

| **Analysis** | **Mplus files in ZIP** | **Verification target** |
| --- | --- | --- |
| Corrected CLPM | 01_clpm_BF.inp to 03_clpm_FS.inp | Cross-lagged paths and fit indices |
| Corrected mediation | 04_mediation_BF.inp to 06_mediation_FS.inp | a-path, b-path, indirect effect, direct effect |
| a-path moderation | 07_apath_moderation_BF.inp to 09_apath_moderation_FS.inp | AA x ML interaction and moderated-mediation index |
| Exploratory b-path moderation | 10_bpath_moderation_BF.inp to 12_bpath_moderation_FS.inp | FNE x ML interaction and conditional indirect effects |
| Phantom-variable sensitivity | 13_phantom_* to 21_phantom_* | Robustness across kappa = .25, .40, .55 |
| Measurement invariance | measurement_invariance_files_from_attachments/ | Configural, metric, scalar CFA syntax/output provided by authors |

**Table S2. Key output: corrected CLPM**

| **Behavior** | **AA T1 -> behavior T2 beta [95% CI]** | **Behavior T1 -> AA T2 beta [95% CI]** | **TLI / CFI** |
| --- | --- | --- | --- |
| Beauty-filter use | .280*** [.189, .371] | .446*** [.339, .553] | .985 / 1.000 |
| Appearance information-seeking | .394*** [.297, .491] | .377*** [.270, .484] | 1.000 / 1.000 |
| Appearance feedback-seeking | .305*** [.210, .400] | .499*** [.399, .599] | .991 / .995 |

**Table S3. Key output: corrected partially longitudinal mediation**

| **Path** | **a-path beta** | **b-path beta** | **Indirect effect** | **95% CI** | **Direct effect beta** |
| --- | --- | --- | --- | --- | --- |
| AA -> FNE -> BF | .370*** | .153* | .057 | [.016, .119] | .258*** |
| AA -> FNE -> IS | .370*** | .249*** | .092 | [.046, .155] | .339*** |
| AA -> FNE -> FS | .370*** | .257*** | .095 | [.049, .159] | .235*** |

**Table S4. Key output: moderation of the a-path**

| **Interaction/indirect path** | **Interaction b** | **SE** | **95% CI** | **p** | **Moderated mediation index 95% CI** |
| --- | --- | --- | --- | --- | --- |
| AA x ML -> FNE -> BF | -.058 | .043 | [-.141, .027] | .174 | [-.030, .002] |
| AA x ML -> FNE -> IS | -.058 | .043 | [-.141, .027] | .174 | [-.040, .005] |
| AA x ML -> FNE -> FS | -.058 | .043 | [-.141, .027] | .174 | [-.042, .005] |

**Table S5. Key output: exploratory moderation of the b-path**

| **Behavior** | **FNE x ML b (SE)** | **p** | **Moderated mediation index [95% CI]** | **Indirect at -1 SD ML** | **Indirect at mean ML** | **Indirect at +1 SD ML** |
| --- | --- | --- | --- | --- | --- | --- |
| Beauty-filter use | 0.133 (0.039) | .001 | 0.049 [.021, .090] | -0.012 [-.076, .049] | 0.043 [-.012, .100] | 0.080 [.031, .157] |
| Appearance information-seeking | 0.036 (0.038) | .342 | 0.013 [-.013, .043] | 0.102 [.036, .188] | 0.101 [.053, .165] | 0.096 [.043, .171] |
| Appearance feedback-seeking | 0.021 (0.036) | .555 | 0.008 [-.017, .035] | 0.110 [.047, .194] | 0.103 [.056, .167] | 0.092 [.044, .164] |

**Table S6. Key output: assumption-dependent phantom-variable sensitivity analysis**

| **Behavior** | **kappa** | **Fixed stable covariance** | **AA -> behavior beta** | **p** | **Behavior -> AA beta** | **p** |
| --- | --- | --- | --- | --- | --- | --- |
| Beauty-filter use | .25 | .115 | .374 | < .001 | .577 | < .001 |
| Beauty-filter use | .40 | .183 | .467 | < .001 | .721 | < .001 |
| Beauty-filter use | .55 | .252 | .623 | < .001 | .962 | < .001 |
| Appearance information-seeking | .25 | .121 | .527 | < .001 | .504 | < .001 |
| Appearance information-seeking | .40 | .193 | .658 | < .001 | .630 | < .001 |
| Appearance information-seeking | .55 | .265 | .878 | < .001 | .840 | < .001 |
| Appearance feedback-seeking | .25 | .117 | .408 | < .001 | .665 | < .001 |
| Appearance feedback-seeking | .40 | .187 | .510 | < .001 | .831 | < .001 |
| Appearance feedback-seeking | .55 | .257 | .680 | < .001 | 1.108 | < .001 |

**Table S7. Longitudinal measurement invariance fit indices**

| **Construct** | **Model** | **chi-square** | **df** | **CFI** | **TLI** | **RMSEA** | **SRMR** | **Delta CFI** | **Delta RMSEA** |
| --- | --- | --- | --- | --- | --- | --- | --- | --- | --- |
| AA | Configural | 673.07 | 335 | .949 | .943 | .055 | .036 | -- | -- |
| AA | Metric | 716.47 | 348 | .945 | .940 | .056 | .054 | -.005 | .001 |
| AA | Scalar | 801.68 | 361 | .934 | .931 | .060 | .055 | -.011 | .004 |
| BF | Configural | 26.00 | 29 | 1.000 | 1.002 | .000 | .017 | -- | -- |
| BF | Metric | 35.88 | 33 | .999 | .998 | .016 | .033 | -.001 | .016 |
| BF | Scalar | 41.17 | 37 | .998 | .998 | .018 | .033 | -.001 | .002 |
| IS | Configural | 78.67 | 29 | .976 | .962 | .071 | .031 | -- | -- |
| IS | Metric | 80.70 | 33 | .977 | .968 | .065 | .033 | .001 | -.006 |
| IS | Scalar | 90.35 | 37 | .974 | .968 | .065 | .033 | -.003 | -.000 |
| FS | Configural | 38.06 | 15 | .987 | .975 | .067 | .030 | -- | -- |
| FS | Metric | 44.01 | 18 | .985 | .977 | .065 | .039 | -.002 | -.002 |
| FS | Scalar | 60.50 | 21 | .977 | .970 | .075 | .039 | -.008 | .009 |

Interpretive note. The phantom-variable models are assumption-dependent sensitivity analyses, not formally identified two-wave RI-CLPMs. They impose fixed stable-component variances and covariances and should not be interpreted as establishing within-person reciprocal processes or as fully removing stable between-person differences.

# Complete Mplus Syntax

The following sections reproduce the complete Mplus input syntax for the corrected CLPM, mediation, moderation, and phantom-variable sensitivity analyses.

## 01_clpm_BF.inp

TITLE:
 Corrected CLPM: appearance anxiety and beauty-filter use.

DATA:
 FILE = appearance_panel_analysis.dat;

VARIABLE:
 NAMES = id age education bmi AA_T1 FNE_T1 ML_T1 BF_T1 IS_T1 FS_T1 AA_T2 BF_T2 IS_T2 FS_T2;
 USEVARIABLES = age education bmi AA_T1 BF_T1 AA_T2 BF_T2;
 MISSING = ALL (-999);

ANALYSIS:
 ESTIMATOR = MLR;

MODEL:
 AA_T2 ON AA_T1 BF_T1 age education bmi;
 BF_T2 ON BF_T1 AA_T1 age education bmi;
 AA_T1 WITH BF_T1;
 AA_T2 WITH BF_T2;

OUTPUT:
 STANDARDIZED CINTERVAL TECH1;

## 02_clpm_IS.inp

TITLE:
 Corrected CLPM: appearance anxiety and appearance information-seeking.

DATA:
 FILE = appearance_panel_analysis.dat;

VARIABLE:
 NAMES = id age education bmi AA_T1 FNE_T1 ML_T1 BF_T1 IS_T1 FS_T1 AA_T2 BF_T2 IS_T2 FS_T2;
 USEVARIABLES = age education bmi AA_T1 IS_T1 AA_T2 IS_T2;
 MISSING = ALL (-999);

ANALYSIS:
 ESTIMATOR = MLR;

MODEL:
 AA_T2 ON AA_T1 IS_T1 age education bmi;
 IS_T2 ON IS_T1 AA_T1 age education bmi;
 AA_T1 WITH IS_T1;
 AA_T2 WITH IS_T2;

OUTPUT:
 STANDARDIZED CINTERVAL TECH1;

## 03_clpm_FS.inp

TITLE:
 Corrected CLPM: appearance anxiety and appearance feedback-seeking.

DATA:
 FILE = appearance_panel_analysis.dat;

VARIABLE:
 NAMES = id age education bmi AA_T1 FNE_T1 ML_T1 BF_T1 IS_T1 FS_T1 AA_T2 BF_T2 IS_T2 FS_T2;
 USEVARIABLES = age education bmi AA_T1 FS_T1 AA_T2 FS_T2;
 MISSING = ALL (-999);

ANALYSIS:
 ESTIMATOR = MLR;

MODEL:
 AA_T2 ON AA_T1 FS_T1 age education bmi;
 FS_T2 ON FS_T1 AA_T1 age education bmi;
 AA_T1 WITH FS_T1;
 AA_T2 WITH FS_T2;

OUTPUT:
 STANDARDIZED CINTERVAL TECH1;

## 04_mediation_BF.inp

TITLE:
 Corrected partially longitudinal mediation: AA -> FNE -> beauty-filter use.

DATA:
 FILE = appearance_panel_analysis.dat;

VARIABLE:
 NAMES = id age education bmi AA_T1 FNE_T1 ML_T1 BF_T1 IS_T1 FS_T1 AA_T2 BF_T2 IS_T2 FS_T2;
 USEVARIABLES = age education bmi AA_T1 FNE_T1 BF_T1 BF_T2;
 MISSING = ALL (-999);

ANALYSIS:
 ESTIMATOR = MLR;
 BOOTSTRAP = 5000;

MODEL:
 FNE_T1 ON AA_T1 (a) age education bmi;
 BF_T2 ON FNE_T1 (b) AA_T1 (cprime) BF_T1 age education bmi;

MODEL CONSTRAINT:
 NEW(indirect);
 indirect = a*b;

OUTPUT:
 STANDARDIZED CINTERVAL(BCBOOTSTRAP) TECH1;

## 05_mediation_IS.inp

TITLE:
 Corrected partially longitudinal mediation: AA -> FNE -> appearance information-seeking.

DATA:
 FILE = appearance_panel_analysis.dat;

VARIABLE:
 NAMES = id age education bmi AA_T1 FNE_T1 ML_T1 BF_T1 IS_T1 FS_T1 AA_T2 BF_T2 IS_T2 FS_T2;
 USEVARIABLES = age education bmi AA_T1 FNE_T1 IS_T1 IS_T2;
 MISSING = ALL (-999);

ANALYSIS:
 ESTIMATOR = MLR;
 BOOTSTRAP = 5000;

MODEL:
 FNE_T1 ON AA_T1 (a) age education bmi;
 IS_T2 ON FNE_T1 (b) AA_T1 (cprime) IS_T1 age education bmi;

MODEL CONSTRAINT:
 NEW(indirect);
 indirect = a*b;

OUTPUT:
 STANDARDIZED CINTERVAL(BCBOOTSTRAP) TECH1;

## 06_mediation_FS.inp

TITLE:
 Corrected partially longitudinal mediation: AA -> FNE -> appearance feedback-seeking.

DATA:
 FILE = appearance_panel_analysis.dat;

VARIABLE:
 NAMES = id age education bmi AA_T1 FNE_T1 ML_T1 BF_T1 IS_T1 FS_T1 AA_T2 BF_T2 IS_T2 FS_T2;
 USEVARIABLES = age education bmi AA_T1 FNE_T1 FS_T1 FS_T2;
 MISSING = ALL (-999);

ANALYSIS:
 ESTIMATOR = MLR;
 BOOTSTRAP = 5000;

MODEL:
 FNE_T1 ON AA_T1 (a) age education bmi;
 FS_T2 ON FNE_T1 (b) AA_T1 (cprime) FS_T1 age education bmi;

MODEL CONSTRAINT:
 NEW(indirect);
 indirect = a*b;

OUTPUT:
 STANDARDIZED CINTERVAL(BCBOOTSTRAP) TECH1;

## 07_apath_moderation_BF.inp

TITLE:
 Moderated mediation, a-path moderation: AA x media literacy -> FNE -> beauty-filter use.

DATA:
 FILE = appearance_panel_analysis.dat;

VARIABLE:
 NAMES = id age education bmi AA_T1 FNE_T1 ML_T1 BF_T1 IS_T1 FS_T1 AA_T2 BF_T2 IS_T2 FS_T2;
 USEVARIABLES = age education bmi AA_T1 ML_T1 AAxML FNE_T1 BF_T1 BF_T2;
 MISSING = ALL (-999);

DEFINE:
 STANDARDIZE AA_T1;
 CENTER ML_T1 (GRANDMEAN);
 AAxML = AA_T1*ML_T1;

ANALYSIS:
 ESTIMATOR = MLR;
 BOOTSTRAP = 5000;

MODEL:
 FNE_T1 ON AA_T1 (a1) ML_T1 (a2) AAxML (a3) age education bmi;
 BF_T2 ON FNE_T1 (b) AA_T1 (cprime) BF_T1 age education bmi;

MODEL CONSTRAINT:
 NEW(indirect modmed_index);
 indirect = a1*b;
 modmed_index = a3*b;

OUTPUT:
 STANDARDIZED CINTERVAL(BCBOOTSTRAP) TECH1;

## 08_apath_moderation_IS.inp

TITLE:
 Moderated mediation, a-path moderation: AA x media literacy -> FNE -> appearance information-seeking.

DATA:
 FILE = appearance_panel_analysis.dat;

VARIABLE:
 NAMES = id age education bmi AA_T1 FNE_T1 ML_T1 BF_T1 IS_T1 FS_T1 AA_T2 BF_T2 IS_T2 FS_T2;
 USEVARIABLES = age education bmi AA_T1 ML_T1 AAxML FNE_T1 IS_T1 IS_T2;
 MISSING = ALL (-999);

DEFINE:
 STANDARDIZE AA_T1;
 CENTER ML_T1 (GRANDMEAN);
 AAxML = AA_T1*ML_T1;

ANALYSIS:
 ESTIMATOR = MLR;
 BOOTSTRAP = 5000;

MODEL:
 FNE_T1 ON AA_T1 (a1) ML_T1 (a2) AAxML (a3) age education bmi;
 IS_T2 ON FNE_T1 (b) AA_T1 (cprime) IS_T1 age education bmi;

MODEL CONSTRAINT:
 NEW(indirect modmed_index);
 indirect = a1*b;
 modmed_index = a3*b;

OUTPUT:
 STANDARDIZED CINTERVAL(BCBOOTSTRAP) TECH1;

## 09_apath_moderation_FS.inp

TITLE:
 Moderated mediation, a-path moderation: AA x media literacy -> FNE -> appearance feedback-seeking.

DATA:
 FILE = appearance_panel_analysis.dat;

VARIABLE:
 NAMES = id age education bmi AA_T1 FNE_T1 ML_T1 BF_T1 IS_T1 FS_T1 AA_T2 BF_T2 IS_T2 FS_T2;
 USEVARIABLES = age education bmi AA_T1 ML_T1 AAxML FNE_T1 FS_T1 FS_T2;
 MISSING = ALL (-999);

DEFINE:
 STANDARDIZE AA_T1;
 CENTER ML_T1 (GRANDMEAN);
 AAxML = AA_T1*ML_T1;

ANALYSIS:
 ESTIMATOR = MLR;
 BOOTSTRAP = 5000;

MODEL:
 FNE_T1 ON AA_T1 (a1) ML_T1 (a2) AAxML (a3) age education bmi;
 FS_T2 ON FNE_T1 (b) AA_T1 (cprime) FS_T1 age education bmi;

MODEL CONSTRAINT:
 NEW(indirect modmed_index);
 indirect = a1*b;
 modmed_index = a3*b;

OUTPUT:
 STANDARDIZED CINTERVAL(BCBOOTSTRAP) TECH1;

## 10_bpath_moderation_BF.inp

TITLE:
 Exploratory moderated mediation, b-path moderation: FNE x media literacy -> beauty-filter use.

DATA:
 FILE = appearance_panel_analysis.dat;

VARIABLE:
 NAMES = id age education bmi AA_T1 FNE_T1 ML_T1 BF_T1 IS_T1 FS_T1 AA_T2 BF_T2 IS_T2 FS_T2;
 USEVARIABLES = age education bmi AA_T1 ML_T1 AAxML FNE_T1 FNExML BF_T1 BF_T2;
 MISSING = ALL (-999);

DEFINE:
 STANDARDIZE AA_T1;
 CENTER ML_T1 FNE_T1 (GRANDMEAN);
 AAxML = AA_T1*ML_T1;
 FNExML = FNE_T1*ML_T1;

ANALYSIS:
 ESTIMATOR = MLR;
 BOOTSTRAP = 5000;

MODEL:
 FNE_T1 ON AA_T1 (a1) ML_T1 (a2) AAxML (a3) age education bmi;
 BF_T2 ON FNE_T1 (b1) ML_T1 (b2) FNExML (b3)
 AA_T1 AAxML BF_T1 age education bmi;

MODEL CONSTRAINT:
 NEW(ind_low ind_mean ind_high modmed_index_b);
 ! Media literacy was mean-centered; conditional effects use +/- 1 SD ML = 1.08.
 ind_low = (a1 + a3*(-1.08))*(b1 + b3*(-1.08));
 ind_mean = a1*b1;
 ind_high = (a1 + a3*(1.08))*(b1 + b3*(1.08));
 modmed_index_b = a1*b3;

OUTPUT:
 STANDARDIZED CINTERVAL(BCBOOTSTRAP) TECH1;

## 11_bpath_moderation_IS.inp

TITLE:
 Exploratory moderated mediation, b-path moderation: FNE x media literacy -> appearance information-seeking.

DATA:
 FILE = appearance_panel_analysis.dat;

VARIABLE:
 NAMES = id age education bmi AA_T1 FNE_T1 ML_T1 BF_T1 IS_T1 FS_T1 AA_T2 BF_T2 IS_T2 FS_T2;
 USEVARIABLES = age education bmi AA_T1 ML_T1 AAxML FNE_T1 FNExML IS_T1 IS_T2;
 MISSING = ALL (-999);

DEFINE:
 STANDARDIZE AA_T1;
 CENTER ML_T1 FNE_T1 (GRANDMEAN);
 AAxML = AA_T1*ML_T1;
 FNExML = FNE_T1*ML_T1;

ANALYSIS:
 ESTIMATOR = MLR;
 BOOTSTRAP = 5000;

MODEL:
 FNE_T1 ON AA_T1 (a1) ML_T1 (a2) AAxML (a3) age education bmi;
 IS_T2 ON FNE_T1 (b1) ML_T1 (b2) FNExML (b3)
 AA_T1 AAxML IS_T1 age education bmi;

MODEL CONSTRAINT:
 NEW(ind_low ind_mean ind_high modmed_index_b);
 ! Media literacy was mean-centered; conditional effects use +/- 1 SD ML = 1.08.
 ind_low = (a1 + a3*(-1.08))*(b1 + b3*(-1.08));
 ind_mean = a1*b1;
 ind_high = (a1 + a3*(1.08))*(b1 + b3*(1.08));
 modmed_index_b = a1*b3;

OUTPUT:
 STANDARDIZED CINTERVAL(BCBOOTSTRAP) TECH1;

## 12_bpath_moderation_FS.inp

TITLE:
 Exploratory moderated mediation, b-path moderation: FNE x media literacy -> appearance feedback-seeking.

DATA:
 FILE = appearance_panel_analysis.dat;

VARIABLE:
 NAMES = id age education bmi AA_T1 FNE_T1 ML_T1 BF_T1 IS_T1 FS_T1 AA_T2 BF_T2 IS_T2 FS_T2;
 USEVARIABLES = age education bmi AA_T1 ML_T1 AAxML FNE_T1 FNExML FS_T1 FS_T2;
 MISSING = ALL (-999);

DEFINE:
 STANDARDIZE AA_T1;
 CENTER ML_T1 FNE_T1 (GRANDMEAN);
 AAxML = AA_T1*ML_T1;
 FNExML = FNE_T1*ML_T1;

ANALYSIS:
 ESTIMATOR = MLR;
 BOOTSTRAP = 5000;

MODEL:
 FNE_T1 ON AA_T1 (a1) ML_T1 (a2) AAxML (a3) age education bmi;
 FS_T2 ON FNE_T1 (b1) ML_T1 (b2) FNExML (b3)
 AA_T1 AAxML FS_T1 age education bmi;

MODEL CONSTRAINT:
 NEW(ind_low ind_mean ind_high modmed_index_b);
 ! Media literacy was mean-centered; conditional effects use +/- 1 SD ML = 1.08.
 ind_low = (a1 + a3*(-1.08))*(b1 + b3*(-1.08));
 ind_mean = a1*b1;
 ind_high = (a1 + a3*(1.08))*(b1 + b3*(1.08));
 modmed_index_b = a1*b3;

OUTPUT:
 STANDARDIZED CINTERVAL(BCBOOTSTRAP) TECH1;

## 13_phantom_BF_kappa_025.inp

TITLE:
 Assumption-dependent phantom-variable sensitivity analysis:
 appearance anxiety and beauty-filter use; kappa = 0.25.
 This model is not a formally identified two-wave RI-CLPM.

DATA:
 FILE = appearance_panel_analysis_standardized.dat;

VARIABLE:
 NAMES = id age education bmi AA_T1 BF_T1 IS_T1 FS_T1 AA_T2 BF_T2 IS_T2 FS_T2;
 USEVARIABLES = age education bmi AA_T1 BF_T1 AA_T2 BF_T2;
 MISSING = ALL (-999);

ANALYSIS:
 ESTIMATOR = MLR;

MODEL:
 RI_A BY AA_T1@1 AA_T2@1;
 RI_B BY BF_T1@1 BF_T2@1;
 wA1 BY AA_T1@1;
 wA2 BY AA_T2@1;
 wB1 BY BF_T1@1;
 wB2 BY BF_T2@1;

 AA_T1@0;
 AA_T2@0;
 BF_T1@0;
 BF_T2@0;
 [RI_A@0 RI_B@0 wA1@0 wA2@0 wB1@0 wB2@0];

 RI_A@0.25;
 RI_B@0.25;
 RI_A WITH RI_B@0.115;

 RI_A WITH wA1@0 wA2@0 wB1@0 wB2@0;
 RI_B WITH wA1@0 wA2@0 wB1@0 wB2@0;

 wA1 WITH wB1;
 wA2 WITH wB2;
 wA2 ON wA1 wB1 age education bmi;
 wB2 ON wB1 wA1 age education bmi;

OUTPUT:
 STANDARDIZED CINTERVAL TECH1;

## 14_phantom_BF_kappa_04.inp

TITLE:
 Assumption-dependent phantom-variable sensitivity analysis:
 appearance anxiety and beauty-filter use; kappa = 0.40.
 This model is not a formally identified two-wave RI-CLPM.

DATA:
 FILE = appearance_panel_analysis_standardized.dat;

VARIABLE:
 NAMES = id age education bmi AA_T1 BF_T1 IS_T1 FS_T1 AA_T2 BF_T2 IS_T2 FS_T2;
 USEVARIABLES = age education bmi AA_T1 BF_T1 AA_T2 BF_T2;
 MISSING = ALL (-999);

ANALYSIS:
 ESTIMATOR = MLR;

MODEL:
 RI_A BY AA_T1@1 AA_T2@1;
 RI_B BY BF_T1@1 BF_T2@1;
 wA1 BY AA_T1@1;
 wA2 BY AA_T2@1;
 wB1 BY BF_T1@1;
 wB2 BY BF_T2@1;

 AA_T1@0;
 AA_T2@0;
 BF_T1@0;
 BF_T2@0;
 [RI_A@0 RI_B@0 wA1@0 wA2@0 wB1@0 wB2@0];

 RI_A@0.40;
 RI_B@0.40;
 RI_A WITH RI_B@0.184;

 RI_A WITH wA1@0 wA2@0 wB1@0 wB2@0;
 RI_B WITH wA1@0 wA2@0 wB1@0 wB2@0;

 wA1 WITH wB1;
 wA2 WITH wB2;
 wA2 ON wA1 wB1 age education bmi;
 wB2 ON wB1 wA1 age education bmi;

OUTPUT:
 STANDARDIZED CINTERVAL TECH1;

## 15_phantom_BF_kappa_055.inp

TITLE:
 Assumption-dependent phantom-variable sensitivity analysis:
 appearance anxiety and beauty-filter use; kappa = 0.55.
 This model is not a formally identified two-wave RI-CLPM.

DATA:
 FILE = appearance_panel_analysis_standardized.dat;

VARIABLE:
 NAMES = id age education bmi AA_T1 BF_T1 IS_T1 FS_T1 AA_T2 BF_T2 IS_T2 FS_T2;
 USEVARIABLES = age education bmi AA_T1 BF_T1 AA_T2 BF_T2;
 MISSING = ALL (-999);

ANALYSIS:
 ESTIMATOR = MLR;

MODEL:
 RI_A BY AA_T1@1 AA_T2@1;
 RI_B BY BF_T1@1 BF_T2@1;
 wA1 BY AA_T1@1;
 wA2 BY AA_T2@1;
 wB1 BY BF_T1@1;
 wB2 BY BF_T2@1;

 AA_T1@0;
 AA_T2@0;
 BF_T1@0;
 BF_T2@0;
 [RI_A@0 RI_B@0 wA1@0 wA2@0 wB1@0 wB2@0];

 RI_A@0.55;
 RI_B@0.55;
 RI_A WITH RI_B@0.253;

 RI_A WITH wA1@0 wA2@0 wB1@0 wB2@0;
 RI_B WITH wA1@0 wA2@0 wB1@0 wB2@0;

 wA1 WITH wB1;
 wA2 WITH wB2;
 wA2 ON wA1 wB1 age education bmi;
 wB2 ON wB1 wA1 age education bmi;

OUTPUT:
 STANDARDIZED CINTERVAL TECH1;

## 16_phantom_IS_kappa_025.inp

TITLE:
 Assumption-dependent phantom-variable sensitivity analysis:
 appearance anxiety and appearance information-seeking; kappa = 0.25.
 This model is not a formally identified two-wave RI-CLPM.

DATA:
 FILE = appearance_panel_analysis_standardized.dat;

VARIABLE:
 NAMES = id age education bmi AA_T1 BF_T1 IS_T1 FS_T1 AA_T2 BF_T2 IS_T2 FS_T2;
 USEVARIABLES = age education bmi AA_T1 IS_T1 AA_T2 IS_T2;
 MISSING = ALL (-999);

ANALYSIS:
 ESTIMATOR = MLR;

MODEL:
 RI_A BY AA_T1@1 AA_T2@1;
 RI_B BY IS_T1@1 IS_T2@1;
 wA1 BY AA_T1@1;
 wA2 BY AA_T2@1;
 wB1 BY IS_T1@1;
 wB2 BY IS_T2@1;

 AA_T1@0;
 AA_T2@0;
 IS_T1@0;
 IS_T2@0;
 [RI_A@0 RI_B@0 wA1@0 wA2@0 wB1@0 wB2@0];

 RI_A@0.25;
 RI_B@0.25;
 RI_A WITH RI_B@0.120;

 RI_A WITH wA1@0 wA2@0 wB1@0 wB2@0;
 RI_B WITH wA1@0 wA2@0 wB1@0 wB2@0;

 wA1 WITH wB1;
 wA2 WITH wB2;
 wA2 ON wA1 wB1 age education bmi;
 wB2 ON wB1 wA1 age education bmi;

OUTPUT:
 STANDARDIZED CINTERVAL TECH1;

## 17_phantom_IS_kappa_04.inp

TITLE:
 Assumption-dependent phantom-variable sensitivity analysis:
 appearance anxiety and appearance information-seeking; kappa = 0.40.
 This model is not a formally identified two-wave RI-CLPM.

DATA:
 FILE = appearance_panel_analysis_standardized.dat;

VARIABLE:
 NAMES = id age education bmi AA_T1 BF_T1 IS_T1 FS_T1 AA_T2 BF_T2 IS_T2 FS_T2;
 USEVARIABLES = age education bmi AA_T1 IS_T1 AA_T2 IS_T2;
 MISSING = ALL (-999);

ANALYSIS:
 ESTIMATOR = MLR;

MODEL:
 RI_A BY AA_T1@1 AA_T2@1;
 RI_B BY IS_T1@1 IS_T2@1;
 wA1 BY AA_T1@1;
 wA2 BY AA_T2@1;
 wB1 BY IS_T1@1;
 wB2 BY IS_T2@1;

 AA_T1@0;
 AA_T2@0;
 IS_T1@0;
 IS_T2@0;
 [RI_A@0 RI_B@0 wA1@0 wA2@0 wB1@0 wB2@0];

 RI_A@0.40;
 RI_B@0.40;
 RI_A WITH RI_B@0.192;

 RI_A WITH wA1@0 wA2@0 wB1@0 wB2@0;
 RI_B WITH wA1@0 wA2@0 wB1@0 wB2@0;

 wA1 WITH wB1;
 wA2 WITH wB2;
 wA2 ON wA1 wB1 age education bmi;
 wB2 ON wB1 wA1 age education bmi;

OUTPUT:
 STANDARDIZED CINTERVAL TECH1;

## 18_phantom_IS_kappa_055.inp

TITLE:
 Assumption-dependent phantom-variable sensitivity analysis:
 appearance anxiety and appearance information-seeking; kappa = 0.55.
 This model is not a formally identified two-wave RI-CLPM.

DATA:
 FILE = appearance_panel_analysis_standardized.dat;

VARIABLE:
 NAMES = id age education bmi AA_T1 BF_T1 IS_T1 FS_T1 AA_T2 BF_T2 IS_T2 FS_T2;
 USEVARIABLES = age education bmi AA_T1 IS_T1 AA_T2 IS_T2;
 MISSING = ALL (-999);

ANALYSIS:
 ESTIMATOR = MLR;

MODEL:
 RI_A BY AA_T1@1 AA_T2@1;
 RI_B BY IS_T1@1 IS_T2@1;
 wA1 BY AA_T1@1;
 wA2 BY AA_T2@1;
 wB1 BY IS_T1@1;
 wB2 BY IS_T2@1;

 AA_T1@0;
 AA_T2@0;
 IS_T1@0;
 IS_T2@0;
 [RI_A@0 RI_B@0 wA1@0 wA2@0 wB1@0 wB2@0];

 RI_A@0.55;
 RI_B@0.55;
 RI_A WITH RI_B@0.264;

 RI_A WITH wA1@0 wA2@0 wB1@0 wB2@0;
 RI_B WITH wA1@0 wA2@0 wB1@0 wB2@0;

 wA1 WITH wB1;
 wA2 WITH wB2;
 wA2 ON wA1 wB1 age education bmi;
 wB2 ON wB1 wA1 age education bmi;

OUTPUT:
 STANDARDIZED CINTERVAL TECH1;

## 19_phantom_FS_kappa_025.inp

TITLE:
 Assumption-dependent phantom-variable sensitivity analysis:
 appearance anxiety and appearance feedback-seeking; kappa = 0.25.
 This model is not a formally identified two-wave RI-CLPM.

DATA:
 FILE = appearance_panel_analysis_standardized.dat;

VARIABLE:
 NAMES = id age education bmi AA_T1 BF_T1 IS_T1 FS_T1 AA_T2 BF_T2 IS_T2 FS_T2;
 USEVARIABLES = age education bmi AA_T1 FS_T1 AA_T2 FS_T2;
 MISSING = ALL (-999);

ANALYSIS:
 ESTIMATOR = MLR;

MODEL:
 RI_A BY AA_T1@1 AA_T2@1;
 RI_B BY FS_T1@1 FS_T2@1;
 wA1 BY AA_T1@1;
 wA2 BY AA_T2@1;
 wB1 BY FS_T1@1;
 wB2 BY FS_T2@1;

 AA_T1@0;
 AA_T2@0;
 FS_T1@0;
 FS_T2@0;
 [RI_A@0 RI_B@0 wA1@0 wA2@0 wB1@0 wB2@0];

 RI_A@0.25;
 RI_B@0.25;
 RI_A WITH RI_B@0.117;

 RI_A WITH wA1@0 wA2@0 wB1@0 wB2@0;
 RI_B WITH wA1@0 wA2@0 wB1@0 wB2@0;

 wA1 WITH wB1;
 wA2 WITH wB2;
 wA2 ON wA1 wB1 age education bmi;
 wB2 ON wB1 wA1 age education bmi;

OUTPUT:
 STANDARDIZED CINTERVAL TECH1;

## 20_phantom_FS_kappa_04.inp

TITLE:
 Assumption-dependent phantom-variable sensitivity analysis:
 appearance anxiety and appearance feedback-seeking; kappa = 0.40.
 This model is not a formally identified two-wave RI-CLPM.

DATA:
 FILE = appearance_panel_analysis_standardized.dat;

VARIABLE:
 NAMES = id age education bmi AA_T1 BF_T1 IS_T1 FS_T1 AA_T2 BF_T2 IS_T2 FS_T2;
 USEVARIABLES = age education bmi AA_T1 FS_T1 AA_T2 FS_T2;
 MISSING = ALL (-999);

ANALYSIS:
 ESTIMATOR = MLR;

MODEL:
 RI_A BY AA_T1@1 AA_T2@1;
 RI_B BY FS_T1@1 FS_T2@1;
 wA1 BY AA_T1@1;
 wA2 BY AA_T2@1;
 wB1 BY FS_T1@1;
 wB2 BY FS_T2@1;

 AA_T1@0;
 AA_T2@0;
 FS_T1@0;
 FS_T2@0;
 [RI_A@0 RI_B@0 wA1@0 wA2@0 wB1@0 wB2@0];

 RI_A@0.40;
 RI_B@0.40;
 RI_A WITH RI_B@0.188;

 RI_A WITH wA1@0 wA2@0 wB1@0 wB2@0;
 RI_B WITH wA1@0 wA2@0 wB1@0 wB2@0;

 wA1 WITH wB1;
 wA2 WITH wB2;
 wA2 ON wA1 wB1 age education bmi;
 wB2 ON wB1 wA1 age education bmi;

OUTPUT:
 STANDARDIZED CINTERVAL TECH1;

## 21_phantom_FS_kappa_055.inp

TITLE:
 Assumption-dependent phantom-variable sensitivity analysis:
 appearance anxiety and appearance feedback-seeking; kappa = 0.55.
 This model is not a formally identified two-wave RI-CLPM.

DATA:
 FILE = appearance_panel_analysis_standardized.dat;

VARIABLE:
 NAMES = id age education bmi AA_T1 BF_T1 IS_T1 FS_T1 AA_T2 BF_T2 IS_T2 FS_T2;
 USEVARIABLES = age education bmi AA_T1 FS_T1 AA_T2 FS_T2;
 MISSING = ALL (-999);

ANALYSIS:
 ESTIMATOR = MLR;

MODEL:
 RI_A BY AA_T1@1 AA_T2@1;
 RI_B BY FS_T1@1 FS_T2@1;
 wA1 BY AA_T1@1;
 wA2 BY AA_T2@1;
 wB1 BY FS_T1@1;
 wB2 BY FS_T2@1;

 AA_T1@0;
 AA_T2@0;
 FS_T1@0;
 FS_T2@0;
 [RI_A@0 RI_B@0 wA1@0 wA2@0 wB1@0 wB2@0];

 RI_A@0.55;
 RI_B@0.55;
 RI_A WITH RI_B@0.259;

 RI_A WITH wA1@0 wA2@0 wB1@0 wB2@0;
 RI_B WITH wA1@0 wA2@0 wB1@0 wB2@0;

 wA1 WITH wB1;
 wA2 WITH wB2;
 wA2 ON wA1 wB1 age education bmi;
 wB2 ON wB1 wA1 age education bmi;

OUTPUT:
 STANDARDIZED CINTERVAL TECH1;
